# Dnajb5 From Antarctic Fish Reveals a Redox‐Sensitive Mechanism Coordinating Muscle Regeneration via mTORC1 and HDAC4

**DOI:** 10.1002/jcsm.70332

**Published:** 2026-07-10

**Authors:** Sun‐Hee Cho, Min‐Kyung Choi, Mi‐Ock Baek, Hyeon‐Hee Son, Binh Thanh Nguyen, Min‐Jung Kang, Il‐Chan Kim, Jin‐Hyoung Kim, Mee‐Sup Yoon

**Affiliations:** ^1^ Department of Health Sciences and Technology, Gachon Advanced Institute for Health Science & Technology Gachon University Incheon Republic of Korea; ^2^ Department of Molecular Medicine Gachon University College of Medicine Incheon Republic of Korea; ^3^ Lee Gil Ya Cancer and Diabetes Institute Gachon University Incheon Republic of Korea; ^4^ Department of Bio‐Medical Science Gachon University College of Medicine Incheon Republic of Korea; ^5^ Center for Advanced Biomolecular Recognition Korea Institute of Science and Technology (KIST) Seoul Republic of Korea; ^6^ Division of Bio‐Medical Science and Technology Korea University of Science and Technology (UST) Daejeon Republic of Korea; ^7^ Division of Life Sciences Korea Polar Research Institute Incheon Republic of Korea; ^8^ Polar Science University of Science and Technology Incheon Republic of Korea

**Keywords:** Antarctic fish, Dnajb5, HDAC4, mitochondrial biogenesis, mTOR, muscle regeneration, myogenesis, *Ppargc1a*

## Abstract

**Background:**

Dnajb5, a member of the heat shock protein family, has not been previously reported to play a role in muscle differentiation. We identify Dnajb5 as a negative regulator of myogenesis via mammalian target of rapamycin (mTOR) and histone deacetylase 4 (HDAC4) signalling, functioning as a central controller of muscle growth and metabolism.

**Methods:**

mTOR‐binding proteins were screened by mass spectrometry in Antarctic fish ( *Notothenia coriiceps* ) muscle extracts. Functional roles of Dnajb5 were examined in C2C12 and primary myoblasts using shRNA‐mediated knockdown. In vivo effects were analysed using a BaCl_2_‐induced muscle injury model in mice (*n* = 6–12 per group).

**Results:**

Mass spectrometry identified Dnajb5 as an mTOR‐binding protein. This interaction was conserved in mammalian cells and weakened by hydrogen peroxide (*p* < 0.05). Dnajb5 knockdown in C2C12 myoblasts selectively increased S6K1 phosphorylation without altering basal Akt phosphorylation. Myogenic differentiation increased, characterized by upregulated *Myog*, *Igf2* and *Ckm* expression (*p* < 0.05) and elevated differentiation index (*p* < 0.05). In vivo, Dnajb5 depletion improved tibialis anterior muscle regeneration, increasing regenerating fibre cross‐sectional area by approximately 26% at Day 5 post‐injury (*p* < 0.05). Dnajb5 knockdown increased *Ppargc1a* expression via release of mTORC1 inhibition and preventing the nuclear retention of HDAC4. Mitochondrial DNA copy number increased 1.3‐fold (*p* < 0.05), and oxidative enzyme activity rose significantly (succinate dehydrogenase‐positive fibres: 2.8‐fold (*p* < 0.01), cytochrome c oxidase‐positive fibres: 1.4‐fold (*p* < 0.05). Grip strength improved by 14% (*p* < 0.05), and rotarod endurance increased by 31% (*p* < 0.01) relative to controls.

**Conclusions:**

Dnajb5 functions as a negative regulator of muscle differentiation and regeneration by coordinating mTORC1 signalling and the HDAC4‐MEF2 axis. These findings reveal a redox‐sensitive dual‐pathway mechanism linking protein synthesis and mitochondrial biogenesis.

## 1 Introduction

Muscle differentiation is a tightly regulated process essential for proper muscle growth and function. This process involves a coordinated network of signalling pathways that respond to various stimuli, including nutrient availability, energy status and growth factors. Among these, the mammalian target of rapamycin (mTOR) has emerged as a key player in regulating muscle cell growth and differentiation. mTOR acts as a central node integrating extracellular signals with intracellular growth pathways to promote cellular processes that are critical for muscle development. Disruptions in mTOR signalling have been implicated in several muscle‐related diseases, underscoring its importance in maintaining muscle mass and function.

mTOR is a serine/threonine protein kinase belonging to the phosphatidylinositol 3‐kinase‐related kinases (PIKKs) family [1]. It forms two distinct protein complexes: mTOR complex 1 (mTORC1) and complex 2 (mTORC2). mTORC1, composed of Raptor, PRAS40, mLST8 and DEPTOR, regulates protein synthesis through downstream effectors p70S6 kinase 1 (S6K1) and eIF4E‐binding protein 1 (4E‐BP1) [2]. mTORC1 maintains muscle mass by balancing anabolism and catabolism in response to nutrients and energy availability [1, 2]. mTOR also controls muscle cell differentiation by regulating insulin‐like growth factor 2 (*Igf2*) transcription through the muscle‐specific enhancer (ME) and modulating MyoD activity, which influences the expression of miR‐1‐follistatin [3, 4].

mTOR signalling is crucial for mitochondrial homeostasis [5, 6]. While short‐term inhibition of mTOR by rapamycin has minimal effects on mitochondrial gene transcription, prolonged treatment reduces expression of key transcriptional regulators including peroxisome proliferator‐activated receptor‐gamma coactivator 1 alpha (*Ppargc1a*) and oestrogen‐related receptor (ERR)‐α, resulting in decreased mitochondrial respiration [7]. This *Ppargc1a* regulation occurs through the transcriptional regulator *Yin Yang 1* (YY1), whose deficiency impairs mitochondrial gene expression and oxygen consumption [7]. mTOR coordinates energy homeostasis by directly regulating ERRα target genes involved in energy metabolism [8].

Environmental stress strongly influences mTOR activity. Antarctic notothenioid fish inhabit waters with extremely low temperatures and unusually high oxygen saturation, conditions that promote reactive oxygen species (ROS) accumulation [9]. Notothenioid fish have evolved specialized adaptations to thrive in these extreme conditions, particularly in muscle metabolism and mitochondrial regulation [10]. These adaptations are essential for maintaining muscle function and energy homeostasis in an environment where traditional muscle regulatory mechanisms may be insufficient [11, 12]. Under these conditions, precise regulation of mTOR activity becomes essential for controlling muscle differentiation, growth and mitochondrial abundance while minimizing energy expenditure. Identifying a specific mTOR regulatory factor in Antarctic fish provides insights into adaptive mechanisms for muscle maintenance in extreme environments.

In this study, we identified DnaJ homologue subfamily member B5 (Dnajb5) as a novel mTOR‐binding factor in Antarctic fish muscle. Functional studies in C2C12 myoblasts and mouse injury model reveal that Dnajb5 acts as a redox‐sensitive negative regulator that coordinates muscle differentiation and mitochondrial biogenesis. Notably, we demonstrate that Dnajb5 exerts these effects through a dual mechanism: direct inhibition of mTORC1 signalling and regulation of the HDAC4‐MEF2 axis. These findings highlight a conserved, stress‐responsive mechanism of mTOR regulation and suggest potential implications for muscle wasting conditions.

## 2 Methods

### 2.1 Cell Culture

C2C12 myoblasts (ATCC, Manassas, VA, USA) were cultured in Dulbecco's Modified Eagle Medium (DMEM) containing 4.5 g/L glucose, 10% Fetal Bovine Serum (FBS), and 1% penicillin–streptomycin. The cells were incubated at 37°C with 5% CO_2_. The cells were plated on 0.2% (w/v) gelatin‐coated tissue culture plates and allowed to grow until fully confluent. Myogenic differentiation was then induced by transferring the cells to a differentiation medium (DMEM supplemented with 2% horse serum). The culture medium was changed daily with a fresh differentiation medium for 2–3 days, following established protocols [13]. For the H_2_O_2_ treatment experiment, cells were treated with 150‐μM H_2_O_2_ for 2 days in growth media.

### 2.2 Primary Myoblast Isolation

Hindlimb skeletal muscles were harvested from 8‐ to 9‐week‐old C57BL/6J male mice and finely minced using sterile scissors. The minced tissue was incubated in muscle dissociation buffer (Ham's F‐10 containing 10% horse serum, 1% penicillin–streptomycin and 0.2% (w/v) collagenase II) for 45 min at 37°C with gentle agitation. Following incubation, the tissue suspension was diluted to 50 mL with wash medium (Ham's F‐10 with 10% horse serum and 1% penicillin–streptomycin) and centrifuged at 1600 × g for 5 min at 4°C. The resulting pellet was resuspended in muscle digestion buffer containing 1.65 U/mL dispase II and 150 U/mL collagenase II, then incubated for 30 min at 37°C. The digested tissue was mechanically dissociated by trituration using a 20‐gauge syringe, diluted to 50 mL with wash medium, and centrifuged at 1600 × g for 5 min at 4°C. The supernatant was aspirated, leaving approximately 10 mL of cell suspension, which was sequentially filtered through 100‐ and 40‐μm cell strainers to remove debris and undigested tissue fragments. The filtered cell suspension was collected and plated onto 100‐mm cell culture dishes for 1 h at 37°C to allow fibroblast attachment (pre‐plating step). After 1 h, the nonadherent cell population enriched in myogenic cells was carefully transferred to ECM‐coated culture plates and maintained in proliferation medium (Ham's F‐10 supplemented with 10% horse serum, 2.5 ng/mL basic fibroblast growth factor (bFGF), and 1% penicillin–streptomycin). For differentiation, confluent myoblasts were switched to differentiation medium (DMEM containing 5% horse serum and 1% penicillin–streptomycin) to induce myotube formation.

### 2.3 Lentiviral shRNA Production and Transduction

Lentiviral shRNA constructs targeting mouse Dnajb5 gene were created using the pLKO.1 puro vector system. The constructs targeted the following sequences: Dnajb5 shRNA#1 (TRCN 0000009549; 5′‐CCTGTGAACAAGGCTGTTCTT‐3′), Dnajb5 shRNA#2 (TRCN0000009553; 5′‐ACCGTGAACATTCCCACCATT‐3′) and a scrambled sequence as a negative control (hereinafter referred to as shControl), following established protocols [14]. Lentiviral particles were produced in HEK293T cells using standard methods [14]. For in vitro experiments, C2C12 cells were transduced with these lentiviruses in a growth medium supplemented with 8 μg/mL polybrene. Following infection, cells were selected with 3 μg/mL puromycin for 5 days. For in vivo experiment, lentivirus particles were concentrated 100‐fold by ultracentrifugation at 90000 × g for 90 min at 4°C with an SW41Ti rotor (Beckman, CA, USA) and resuspended in PBS. Concentrated lentiviral particles (30 μL) were coinjected with BaCl_2_ into the TA muscle for in vivo knockdown experiments.

### 2.4 BaCl_2_‐Induced Muscle Injury Model

Ten‐week‐old male C57BL/6N mice (Orient Bio Inc., Seongnam, Republic of Korea) were housed under standard conditions (20°C–22°C, 12 h light/dark cycle) with ad libitum access to food and water. Mice were randomly assigned to experimental groups (*n* = 6–8 per group). To induce muscle injury, 50 μL of 1.2% (w/v) barium chloride (BaCl_2_) in saline containing concentrated lentiviral particles was injected into the tibialis anterior (TA) muscles [14]. Mice were randomly assigned to two groups, receiving lentiviruses expressing either scrambled shRNA (control group) or Dnajb5 shRNA (knockdown group). On Day 5 post‐injection, mice were euthanized, and TA muscles were harvested. The tissues were embedded in OCT compound (Sakura Finetek, Tokyo, Japan), rapidly frozen in isopentane cooled with liquid nitrogen, and sectioned at 10 μm thickness using a cryostat (HM 520, GMI, USA) for histological and molecular analyses.

### 2.5 Ethical Approval and Animal Care

All animal experiments were performed in accordance with institutional ethical guidelines. The mouse injury study was approved by the Institutional Animal Care and Use Committee of the Lee Gil Ya Cancer and Diabetes Institute, Gachon University (approval numbers: LCDI‐2023‐0057). Fish studies were conducted under the ethical guidelines of the Korea Polar Research Institute (KOPRI), with protocols approved by the KOPRI Ethics Committee (approval numbers: KACC2301‐005, KACC2301‐006).

### 2.6 Cell Lysis, Immunoprecipitation and Western Blot Analysis

To prepare cell lysates, the cells were washed with phosphate‐buffered saline (PBS) to remove residual media and lysed using a lysis buffer (9803, Cell Signaling Technology) with protease and phosphatase inhibitors. The lysates were kept on ice for 30 min to ensure thorough lysis, followed by centrifugation at 13200 rpm for 10 min at 4°C to remove cell debris. For endogenous immunoprecipitation, C2C12 cells were lysed with a lysis buffer containing 40‐mM HEPES buffer (pH 7.4), 120‐mM NaCl, 10‐mM sodium pyrophosphate, 50‐mM NaF, 2‐mM EDTA and 0.3% CHAPS supplemented with protease (#04693159001, Roche) and phosphatase inhibitors (#04906837001, Roche). The lysates were incubated with anti‐mTOR antibody at 4°C overnight. The immune complexes were then captured by incubating with protein G agarose beads (Millipore, Molsheim, France) for 1 h at 4°C, followed by three washes with the lysis buffer to remove non‐specifically bound proteins. All protein samples were boiled in sodium dodecyl sulfate (SDS) sample buffer for 3 min. For western blot analysis, proteins were separated by SDS‐polyacrylamide gel electrophoresis (SDS‐PAGE) and transferred onto polyvinylidene fluoride (PVDF) membranes (MilliporeSigma, Burlington, MA, USA). The membranes were blocked by incubating with 5% (w/v) skim milk in Tris‐buffered saline containing 1% Tween‐20 (TBS‐T) for 30 min at room temperature (around 20°C–25°C) to prevent non‐specific binding. The membranes were then incubated with primary antibodies overnight at 4°C. After washing with TBS‐T three times for 10 min each, membranes were incubated with horseradish peroxidase‐conjugated secondary antibodies (Jackson ImmunoResearch Laboratories, West Grove, PA, USA) for 1 h at room temperature, followed by three washes of 10 min each with TBS‐T. Detection was performed using Immobilon Western Chemiluminescent HRP Substrate (Millipore), and signals were visualized with an iBright 1500 imaging system (Thermo Fisher Scientific, Waltham, MA, USA) [15, 16]. Protein bands were quantified using ImageJ software, with normalization to a housekeeping protein such as tubulin. The activity of mTORC1 and mTORC2 was assessed by the phosphorylation ratio of S6K1 (Thr389) and Akt (Ser473) to their respective total proteins.

### 2.7 Subcellular Fractionation

Cells were washed three times with cold PBS and lysed in 300 μL of cytoplasmic protein extraction buffer containing 5‐mM KCl, 5‐mM HEPES (pH 7.9), 0.05‐mM EGTA, 0.05‐mM EDTA and 0.075% NP‐40, supplemented with protease and phosphatase inhibitors. The lysates were incubated on ice for 30 min, with shaking every 10 min. After centrifugation at 3000 × g for 5 min at 4°C, the cytosolic extract (supernatant) was collected. The remaining nuclear pellet was washed three times with the cytoplasmic extraction buffer to remove cytosolic contaminants. The nuclear pellet was then resuspended in 100 μL of nuclear extraction buffer containing 0.25‐M EDTA, 0.5‐M Tris‐HCl (pH 7.4), 2.5‐M NaCl, 5% sodium deoxycholate, 5% SDS and 5% Triton X‐100, supplemented with protease and phosphatase inhibitors. The suspension was incubated on ice for 30 min with vortexing every 10 min. Nuclear extracts were collected by centrifugation at 13 000 × g for 30 min at 4°C. The resulting supernatant was mixed with SDS sample buffer, boiled for 3 min at 100°C and subjected to SDS‐PAGE and western blot analysis as described above.

### 2.8 Luciferase Reporter Assay

C2C12 cells were transfected with either the H19‐Igf2‐ME‐luciferase reporter plasmid or the *Ppargc1a* promoter reporter constructs, along with a β‐galactosidase vector (as an internal control) using lipofectamine 3000 (Invitrogen, Waltham, MA, USA) according to the manufacturer's protocol. The wild‐type *Ppargc1a* promoter luciferase reporter construct (Addgene, #8887) and the MEF2 binding site mutant (*Ppargc1a*‐MEF2) reporter (Addgene, #8889) were obtained from Addgene (Watertown, MA, USA) [17]. Following transfection, cells were allowed to recover for 24 h before being switched to differentiation medium. The cells were lysed 12 h after the induction of differentiation. Luciferase activity was measured using a Luciferase Assay System (Promega, Madison, WI, USA), and β‐galactosidase activity was simultaneously quantified using the β‐galactosidase Enzyme Assay System (Promega) to normalize for transfection efficiency. Luminescence intensity was obtained using a VICTOR NivoTM Multimode Microplate Reader (PerkinElmer; Waltham, MA, USA). Data are presented as relative luciferase activity (Luciferase/β‐gal ratio) from at least three independent experiments.

### 2.9 Immunocytochemistry and Morphological Analysis of Myotubes

C2C12 cells and primary myoblasts were cultured in 12‐well plates for immunofluorescence. Differentiation was induced for 3 days for C2C12 cells and 2 days for primary myoblasts. After differentiation, the cells were fixed with 10% formaldehyde for 10 min at room temperature and permeabilized with 0.1% Triton X‐100 for 5 min. Blocking was done with 3% bovine serum albumin (BSA) in PBS for 30 min. The cells were incubated overnight at 4°C with an anti–myosin heavy chain (MHC) antibody (MF20, 1:100) in PBS containing 3% BSA. After washing with PBS, the cells were treated with a fluorescein isothiocyanate (FITC)‐conjugated secondary antibody (1:200, Jackson ImmunoResearch Laboratories) for 1 h at room temperature in the dark. Nuclei were stained with 4′,6‐diamidino‐2‐phenylindole (DAPI, Vector Laboratories, Burlingame, CA, USA) for 5 min. Images were captured using a fluorescence microscope (Olympus CKX3 Microscope, Olympus, Tokyo, Japan) with a 10× objective. Quantitative analysis was conducted on the images using ImageJ software to assess differentiation. The differentiation index was calculated as the ratio of nuclei within MHC‐positive myotubes to the total number of nuclei. This parameter was calculated from at least five randomly selected microscopic fields per well. Data from three independent experiments were analysed [13].

### 2.10 Histological and Immunofluorescence Analysis of Muscle Sections

#### 2.10.1 Haematoxylin and Eosin (H&E) Staining

Frozen muscle sections (10 μm) were stained with H&E following the manufacturer's protocol. Five to 10 fields within the injured area were imaged randomly using a Motic Easyscan Digital Slide Scanner (Motic Hong Kong Limited). Cross‐sectional area (CSA) of centrally nucleated regenerating myofibres was quantified using ImageJ software (NIH, Bethesda, MD, USA) to assess muscle regeneration.

#### 2.10.2 Immunofluorescence Staining

Sections were washed with PBS, permeabilized with 0.2% Triton X‐100, and blocked in 1% BSA for 2 h. Primary antibodies were applied overnight at 4°C in PBS containing 1% BSA, including markers for basal lamina (laminin), satellite cells (Pax7), proliferation (Ki67) and early differentiation (myogenin). After washing, sections were incubated with appropriate Alexa Fluor‐conjugated secondary antibodies for 1 h at room temperature and counterstained with DAPI. Imaging was performed using CKX53 (Olympus) microscopes. Image analysis was conducted with ImageJ.

### 2.11 SDH/COX Enzyme Histochemistry

To evaluate mitochondrial respiratory enzyme activity, 10‐μm frozen TA muscle sections were subjected to succinate dehydrogenase (SDH) and cytochrome c oxidase (COX) staining. For SDH staining, sections were incubated with 0.2‐M sodium succinate and nitrotetrazolium blue in 0.2‐M phosphate buffer (pH 7.4) at 37°C for 1–2 h, followed by fixation in 10% formalin. Sections were then rinsed, dehydrated through graded ethanol and xylene, and mounted. For COX staining, sections were incubated with COX incubation solution (COX/SDH Double Histochemistry Kit, VitroView, VB‐3002, Rockville, MD, USA) at room temperature for 2 h in the dark. Images were acquired using the Motic Easyscan system. The number of positive fibres was quantified using ImageJ software with intensity thresholds set at 200 for SDH and 170 for COX staining.

### 2.12 Hindlimb Grip Strength Test

Hindlimb grip strength was measured using a grip strength meter (B.S. Technolab Inc., Gangbuk‐gu, Seoul, Korea). Mice were positioned with their torsos parallel to the grid, allowing both the fore and hind paws to grasp the grid before taking measurements. Each mouse was gently pulled horizontally by its tail parallel to the grid until it lost its grip. The apparatus automatically recorded the peak force exerted during resistance. Measurements were performed in at least five trials per mouse to ensure accuracy (*n* = 12 mice per group). Results were normalized to body weight and expressed as force per gram of body weight.

### 2.13 Rotarod Experiment

Motor coordination and balance were assessed using an accelerating rotarod (BS Technolab Inc., Seoul, Korea). Testing was conducted on Day 5 post‐injury using an acceleration paradigm of 4–40 rpm. The latency to fall from the rod was measured over at least three trials per mouse, with a maximum trial duration of 300 s (*n* = 12 mice per group). Results were normalized to body weight and expressed as latency to fall per gram of body weight.

### 2.14 Statistical Analysis

Statistical analyses were performed using GraphPad Prism version 9.0 (GraphPad Software, San Diego, CA, USA). Data are presented as mean ± standard deviation (SD). For in vitro studies, data represent results from at least three independent experiments. For in vivo experiments, sample sizes were determined to ensure sufficient statistical power (*n* = 6–12 mice per group). For comparisons between two groups, a two‐tailed unpaired Student's *t* test was used. For comparisons among three or more groups, one‐way analysis of variance (ANOVA) followed by Tukey's post hoc test was performed. For experiments involving two independent variables, two‐way ANOVA followed by Tukey's multiple comparisons test was used. Statistical significance was defined as *p* < 0.05, and significant results are indicated in the figures by asterisks (*p < 0.05, **p < 0.01, ***p < 0.001).

## 3 Results

### 3.1 Dnajb5 Is Identified as a Novel mTOR‐Binding Protein in Antarctic Fish Muscle

To identify mTOR‐binding proteins in Antarctic fish muscle, we performed immunoprecipitation followed by mass spectrometry analysis. We first validated the specificity of commercially available mTOR antibodies for fish proteins, as cross‐species recognition could not be assumed. While increasing Raptor antibody concentrations did not significantly enhance mTOR recovery (Figure S1a), higher mTOR antibody concentrations proportionally increased mTOR immunoprecipitation yield (Figure S1b), confirming the suitability of the antibody for IP. Using these optimized conditions, we successfully isolated mTOR complexes from the muscles of both Antarctic fish ( *Notothenia coriiceps* ) and pufferfish ( *Takifugu obscurus* ), as verified by western blot detection of mTOR and Raptor (Figure S1c,d). Liquid chromatography–tandem mass spectrometry (LC‐MS/MS) analysis of mTOR immunoprecipitates identified 56 proteins across both species, with 18 proteins unique to *N. coriiceps* and 3 specific to *T. obscurus* (Figure S2a and Table S1). Among proteins with high statistical confidence (false discovery rate [FDR] < 1%), we identified DnaJ homologue subfamily member B5 (Dnajb5), heterogeneous nuclear ribonucleoprotein C‐like and GELS as potential mTOR‐binding factors. Dnajb5 emerged as the most promising candidate based on its established high expression in muscle tissue (Figure S2b and Table S1) and previous associations with cardiac regeneration [18, 19]. RT‐PCR analysis confirmed the expression of both *mtor* and *dnajb5* transcripts in *N. coriiceps* muscle tissue (Figure S2c).

### 3.2 Dnajb5‐mTOR Interaction Is Conserved in Mammalian Cells and Modulated by Oxidative Stress

**FIGURE 1:**
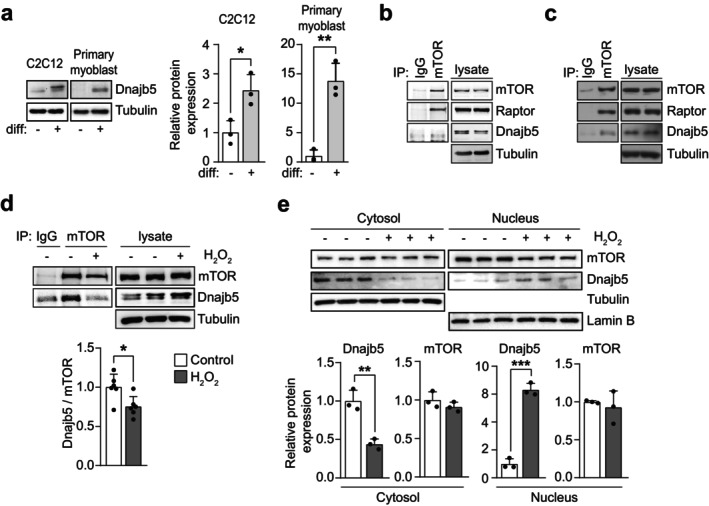
Dnajb5 interacts with mTOR in C2C12 and primary myoblasts, and this interaction is redox‐sensitive. (a) Western blot analysis of Dnajb5 expression in C2C12 cells and primary myoblasts before (−) and after (+) induction of differentiation. Differentiation was induced for 3 days in C2C12 cells and 2 days in primary myoblasts (*n* = 3). Quantification of Dnajb5 protein levels (expressed as a fold change) is shown on the right. Data are presented as mean ± SD; *p < 0.05, **p < 0.01 versus undifferentiated control. (b,c) Coimmunoprecipitation (IP) of endogenous mTOR with Dnajb5 in (b) undifferentiated and (c) differentiated C2C12 cells. Lysates were immunoprecipitated with anti‐mTOR antibody or IgG control, followed by western blot analysis of mTOR, Raptor and Dnajb5. (d) IP with anti‐mTOR antibody in C2C12 cells treated with or without 150‐μM H_2_O_2_ for 2 days. The graph shows the quantification of Dnajb5 bound to mTOR (Dnajb5/mTOR ratio) (expressed as a fold change) (*n* = 6). *p < 0.05. (e) Western blot analysis of cytosolic and nuclear fractions from C2C12 cells treated with or without H_2_O_2_. Dnajb5 and mTOR levels were analysed (*n* = 3). Tubulin and Lamin B served as loading controls for the cytosolic and nuclear fractions, respectively. Graphs show the relative protein quantification (expressed as a fold change). Data are presented as mean ± SD, **p < 0.01, ***p < 0.001.

To validate the Dnajb5‐mTOR interaction in a mammalian system, we examined their relationship in C2C12 mouse myoblasts and primary myoblasts. Dnajb5 expression increased significantly during myogenic differentiation, with protein levels elevated in differentiated cells compared with undifferentiated myoblasts (Figure 1a). Specific detection of Dnajb5 was confirmed by comparing the expression pattern in C2C12 cells with primary myoblasts, which showed a consistent single specific band (Figure 1a). Accordingly, mRNA expression of *Dnajb5* showed a 3.8‐fold upregulation after 2 days of differentiation (Figure S3a). This differentiation‐associated upregulation suggested a functional role for Dnajb5 in myogenesis. Consistent with this, endogenous coimmunoprecipitation experiments confirmed that Dnajb5 associates with mTOR in C2C12 cells both before and after differentiation (Figure 1b,c), indicating that this interaction is independent of differentiation status. We further validated this interaction using Flag‐tagged Dnajb5 overexpressed in HEK293 cells (Figure S3b). These results establish that the Dnajb5 and mTOR interaction is conserved from Antarctic fish to mammalian systems. Since Antarctic environments are characterized by high reactive oxygen species (ROS) levels, we investigated whether oxidative stress affects the Dnajb5‐mTOR interaction. Quantitative analysis of endogenous immunoprecipitates revealed that exposure to hydrogen peroxide (H_2_O_2_) significantly reduced the interaction between Dnajb5 and mTOR (Figure 1d). Furthermore, subcellular fractionation assays showed that oxidative stress induced the nuclear accumulation of Dnajb5, while mTOR remained predominantly cytosolic (Figure 1e). These results suggest that this protein complex is susceptible to redox regulation, leading to the spatial separation of Dnajb5 from mTOR under oxidative stress.

### 3.3 Dnajb5 Negatively Regulates mTOR Activity in C2C12 Cells

**FIGURE 2:**
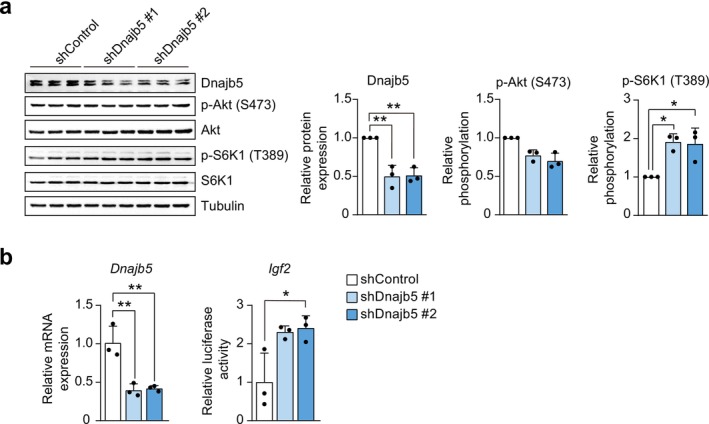
Dnajb5 depletion selectively enhances mTORC1 signalling in C2C12 myoblasts. (a) Western blot analysis of Dnajb5, Akt, p‐Akt (S473), S6K1 and p‐S6K1 (T389) in C2C12 cells transduced with shControl or Dnajb5 shRNAs (shDnajb5 #1 and #2). Tubulin served as a loading control. The graphs show the quantification of band intensities expressed as a fold change relative to shControl (*n* = 3). (b) Luciferase assay of *Igf2* muscle enhancer (ME) reporter activity (expressed as a fold change) in shControl‐ and shDnajb5‐transduced cells after 12 h of differentiation (*n* = 3). Data are presented as mean ± SD. Statistical significance was determined by an unpaired *t* test. *p < 0.05, **p < 0.01.

To determine the functional role of Dnajb5 in mTOR signalling, we used lentiviral short hairpin RNA (shRNA) to knockdown Dnajb5 expression in C2C12 cells. We then assessed mTOR activity by measuring the phosphorylation of downstream effectors: S6K1 (Thr389) for mTORC1 activity and Akt (Ser473) for mTORC2 activity. Dnajb5 knockdown was confirmed by western blot analysis, showing effective reduction in protein levels (Figure 2a). Under basal growth conditions, Dnajb5 depletion selectively enhanced mTORC1 signalling, as evidenced by significantly increased S6K1 phosphorylation at Thr389, while Akt phosphorylation at Ser473 remained unchanged (Figure 2a). This differential effect indicates that Dnajb5 specifically inhibits mTORC1 rather than mTORC2 activity in undifferentiated myoblasts. Notably, when cells were subjected to acute serum stimulation with 10% FBS after serum starvation, Dnajb5 knockdown did not significantly affect the magnitude of mTOR activation (Figure S4), suggesting that Dnajb5 primarily regulates basal mTOR activity rather than acute growth factor‐induced responses. To validate this finding functionally, we examined the effect of Dnajb5 knockdown on *Igf2* muscle enhancer (ME) activity, a known downstream target of mTOR signalling in muscle differentiation. Dnajb5‐depleted cells showed significantly increased *Igf2* ME reporter activity compared with control cells during differentiation (Figure 2b), confirming that Dnajb5 acts as a negative regulator of mTOR‐mediated transcriptional programmes in myogenesis.

### 3.4 Dnajb5 Knockdown Enhances Muscle Differentiation In Vitro

**FIGURE 3:**
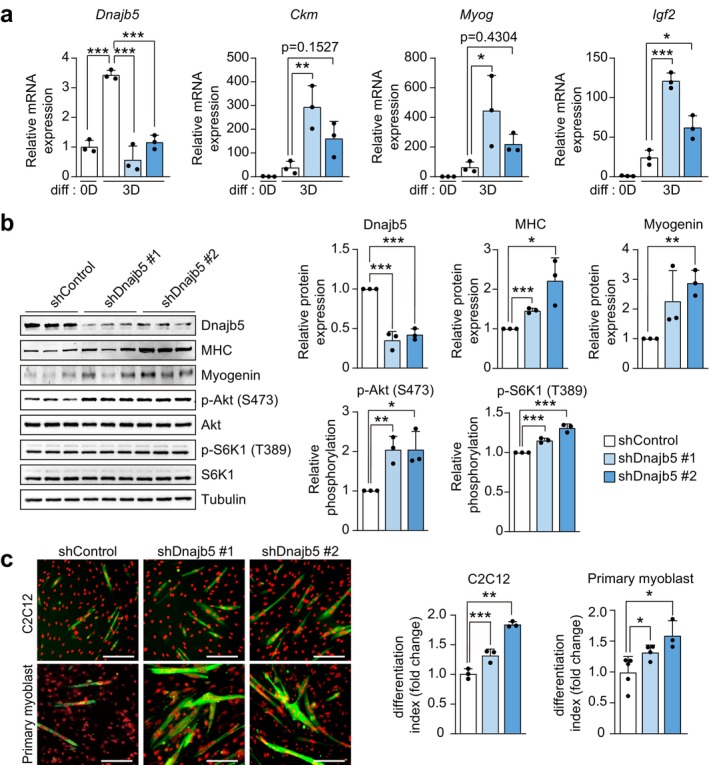
Dnajb5 knockdown promotes myogenic differentiation in C2C12 cells and primary myoblasts. (a) Relative mRNA expression (expressed as a fold change) of *Dnajb5*, *Ckm*, *Igf2* and *Myog* in shControl‐ and shDnajb5‐transduced cells at Day 0 (0D) and Day 3 (3D) of differentiation (*n* = 3). (b) Western blot analysis of Dnajb5, MHC, myogenin, Akt, p‐Akt (S473), S6K1 and p‐S6K1 (T389) in shControl‐ and shDnajb5‐transduced cells after 3 days of differentiation. Tubulin served as a loading control. Band intensities were quantified using ImageJ and expressed as a fold change relative to shControl (right panels) (*n* = 3). (c) Representative immunofluorescence images of MHC (green) and nuclei (red) in shControl‐ and shDnajb5‐transduced cells. Images were acquired after 3 days of differentiation for C2C12 cells and 2 days for primary myoblasts. Scale bars: 50 μm. Quantification of the differentiation index, expressed as a fold change relative to shControl, is shown on the right for both C2C12 and primary cells (*n* = 3–5). Data are presented as mean ± SD. Statistical significance was determined by one‐way ANOVA followed by Tukey's post hoc test for comparisons among three groups (shControl, shDnajb5 #1 and shDnajb5 #2) and unpaired *t* test for two‐group comparisons (if applicable). *p < 0.05, **p < 0.01, ***p < 0.001.

To assess the functional role of Dnajb5 in muscle differentiation, we analysed the expression of differentiation markers following Dnajb5 knockdown in C2C12 cells. Dnajb5 depletion significantly upregulated mRNA levels of key differentiation markers including *Ckm*, *Myog* and *Igf2* compared with shControl (Figure 3a). Consistent with enhanced mTOR signalling, Dnajb5 knockdown also increased phosphorylation of both S6K1 (Thr389) and Akt (Ser473) during differentiation. At the protein level, differentiation markers myosin heavy chain (MHC) and myogenin were significantly elevated in Dnajb5‐depleted cells (Figure 3b). To evaluate morphological changes, we performed immunofluorescence analysis. Differentiation was induced for 3 days in C2C12 cells and 2 days in primary myoblasts. Notably, consistent with the observations in C2C12 cells, Dnajb5 knockdown in primary myoblasts also exhibited increased numbers of myofibres positive for MHC, with notably thicker and longer myofibres compared with shControl (Figure 3c). Quantitative analysis revealed significant increases in differentiation index in Dnajb5‐depleted C2C12 and primary myoblast cultures (Figure 3c), indicating that Dnajb5 depletion robustly enhances myoblast differentiation.

### 3.5 Dnajb5 Knockdown Improves Muscle Regeneration Capacity Following Injury

**FIGURE 4:**
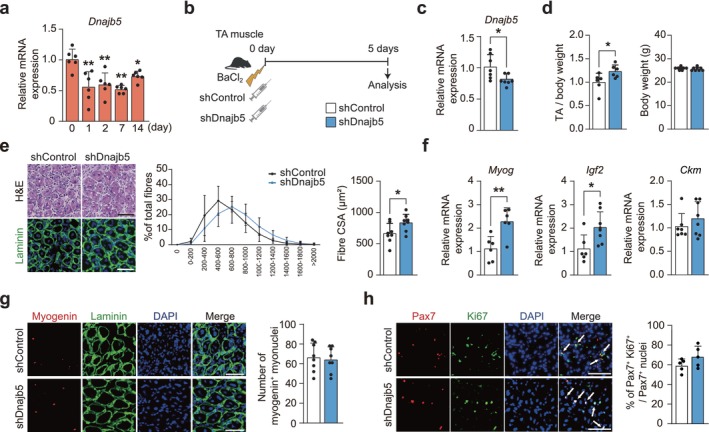
Dnajb5 knockdown promotes myofibre hypertrophy and early myogenic gene expression during regeneration. (a) Time course of *Dnajb5* mRNA expression (expressed as a fold change) during muscle regeneration following BaCl_2_‐induced injury (Days 0, 1, 2, 7 and 14 post‐injury) (*n* = 6). Note that *Dnajb5* levels remain suppressed during the regeneration phase. *p < 0.05, **p < 0.01 versus Day 0. (b) Experimental scheme for Dnajb5 knockdown in TA muscle. BaCl_2_ injury and shRNA (shControl or shDnajb5) were coinjected simultaneously, followed by analysis after 5 days. (c) Dnajb5 knockdown confirmation by qRT‐PCR analysis (expressed as a fold change) 5 days after shRNA treatment (*n* = 7). *p < 0.05. (d) Normalized TA muscle weight (TA/body weight) (expressed as a fold change relative to control) and total body weight measured at 5 days post‐injury (*n* = 6–8). (e) Representative H&E staining (upper panels, Scale bars: 60 μm) and immunofluorescence images (lower panels, Scale bars: 50 μm) of regenerating TA muscle sections. Immunofluorescence shows muscle fibres outlined by laminin (green) and nuclei (DAPI, blue). Fibre size distribution analysis showing percentage of total fibres across different cross‐sectional areas (CSA) (0–200 to > 2000 μm^2^) and average fibre CSA (*n* = 8). *p < 0.05. (f) Relative mRNA expression (expressed as a fold change) of myogenic markers (*Myog*, *Igf2* and *Ckm*) in injured TA muscle 5 days after shRNA treatment (*n* = 6–8). *p < 0.05. (g) Representative immunofluorescence images and quantification of myogenin‐positive myonuclei (red: myogenin, green: laminin, blue: DAPI) (*n* = 8). Scale bars: 50 μm. (h) Representative immunofluorescence images and quantification of colocalization of satellite cell and proliferation markers (red: Pax7, green: Ki67, blue: DAPI). Scale bars: 50 μm. The graph shows the percentage of Pax7^+^Ki67^+^ proliferating satellite cells relative to Pax7^+^ nuclei (*n* = 5). White arrows indicate Pax7^+^Ki67^+^ cells. Data are presented as mean ± SD. Individual data points represent biological replicates. Statistical significance was determined by an unpaired *t* test (two groups). *p < 0.05, **p < 0.01.

To examine the physiological relevance of Dnajb5 in muscle biology, we used a well‐established barium chloride (BaCl_2_)–induced muscle injury model [13, 14]. Dnajb5 mRNA expression was significantly downregulated following injury and remained suppressed through Day 7 post‐injury, before partially recovering by Day 14 (Figure 4a). This temporal expression pattern suggests a negative regulatory role in the regenerative response. We performed loss‐of‐function studies by coinjecting *Dnajb5* shRNA with BaCl_2_ into tibialis anterior (TA) muscles (Figure 4b). Effective knockdown was confirmed by qRT‐PCR analysis, which showed a significant reduction in *Dnajb5* mRNA levels (Figure 4c). Notably, *Dnajb5* depletion significantly increased the normalized wet weight of injured TA muscles (TA weight/body weight ratio), whereas total body weights remained unchanged (Figure 4d). Histological analysis revealed that Dnajb5 depletion significantly enhanced muscle regeneration, as evidenced by significantly larger regenerating myofibres during the early regenerative period compared with control (average fibre cross‐sectional area increased by ~26%) (Figure 4e). However, the total fibre numbers remained unchanged (Figure S5). Molecular analysis revealed selective upregulation of early myogenic markers (*Myog* and *Igf2*) while late marker *Ckm* remained unchanged, suggesting that Dnajb5 specifically regulates early differentiation stages (Figure 4f). Despite significant transcriptional upregulation of myogenic markers, the number of myogenin^+^ myonuclei remained unchanged (Figure 4g), indicating that transcriptional activation does not necessarily translate to increased cellular differentiation. Similarly, the population of Pax7^+^/Ki67^+^ proliferating satellite cells showed only modest, non‐significant increases (Figure 4h). These findings suggest that Dnajb5 knockdown primes early myogenic programs but does not robustly enhance satellite cell activation or the number of differentiated cells in vivo.

### 3.6 Dnajb5 Depletion Enhances Mitochondrial Biogenesis via Dual mTORC1 and HDAC4‐MEF2 Signalling Pathways

**FIGURE 5:**
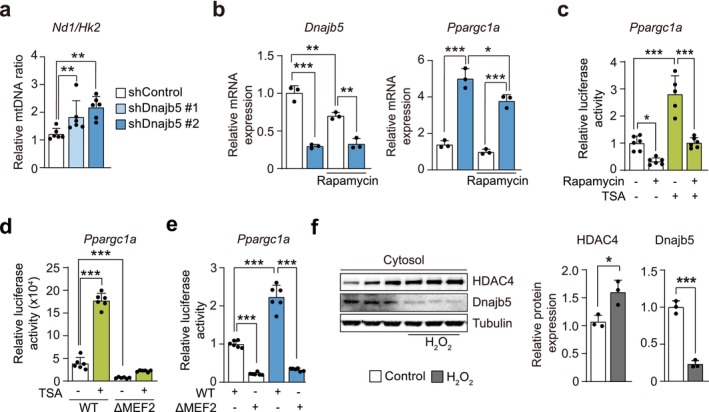
Dnajb5 knockdown promotes mitochondrial biogenesis via dual mTORC1 and HDAC4‐MEF2 signalling pathways. (a) Mitochondrial DNA copy number (ratio of *Nd1* to *Hk2*) (expressed as a fold change) in C2C12 cells at 2 days of differentiation following shControl or shDnajb5 knockdown (*n* = 6). (b) Relative mRNA expression (expressed as a fold change) of *Dnajb5* and *Ppargc1a* in C2C12 cells treated with or without rapamycin during 2 days of C2C12 cell differentiation (*n* = 3). (c) Relative *Ppargc1a* promoter luciferase activity (expressed as a fold change) in C2C12 cells treated with rapamycin and/or Trichostatin A (TSA) (*n* = 5–6). (d) Relative luciferase activity of wild‐type (WT) or MEF2 binding site mutant (ΔMEF2) *Ppargc1a* promoter constructs treated with TSA (*n* = 6). (e) Relative luciferase activity (expressed as a fold change) of WT or ΔMEF2 *Ppargc1a* promoter constructs following shControl or shDnajb5 knockdown (*n* = 6). (f) Western blot analysis of cytosolic fractions from C2C12 cells treated with or without H_2_O_2_ (150 μM) for 2 days (quantification expressed as a fold change) (*n* = 3). Tubulin served as loading controls for cytosolic fractions. Data are presented as mean ± SD. Individual data points represent biological replicates. Statistical significance was determined by two‐way ANOVA followed by Tukey's multiple comparisons test for experiments involving two variables (b,d,e), one‐way ANOVA with Tukeys post hoc test for multiple group comparisons (c), and unpaired *t* test for two‐group comparisons (a,f). *p < 0.05, **p < 0.01, ***p < 0.001.

Given mTOR's established role in mitochondrial biogenesis, we examined whether Dnajb5 affects mitochondrial function. Dnajb5 knockdown substantially increased *Ppargc1a* expression, a master regulator of mitochondrial biogenesis (Figure S6). This transcriptional upregulation correlated with increased mitochondrial DNA (mtDNA) content in C2C12 cells (Figure 5a). Importantly, rapamycin treatment attenuated the Dnajb5 knockdown‐induced *Ppargc1a* upregulation but failed to abolish it completely (Figure 5b). This partial inhibition suggested the involvement of an alternative, mTOR‐independent pathway. Previous studies have shown that MEF2 family transcription factors regulate *Ppargc1a* expression and that their activity is normally repressed by Class IIa HDACs [17]. Notably, Dnajb5 was previously shown to interact with HDAC4, a key repressor of MEF2, and this interaction was dissociated under oxidative stress [20]. Therefore, we hypothesized that Dnajb5 might also regulate *Ppargc1a* via the HDAC4‐MEF2 axis in an mTOR‐independent manner. To test this, we treated cells with the HDAC inhibitor Trichostatin A (TSA) and rapamycin. TSA treatment significantly increased *Ppargc1a* promoter activity (Figure 5c). Importantly, when rapamycin and TSA were cotreated, rapamycin decreased the TSA‐induced activity but failed to abolish it completely (Figure 5c). This effect strongly suggests that the HDAC‐mediated regulation operates independently of the mTOR pathway. Furthermore, the upregulation of *Ppargc1a* induced by either TSA or Dnajb5 knockdown was significantly blunted when the MEF2 binding site on the *Ppargc1a* promoter was mutated (Figure 5d,e). This confirms that Dnajb5 exerts its repressive effect, at least in part, through the MEF2 transcription factor. Mechanistically, we investigated the effect of oxidative stress on the subcellular localization of these regulators. Cellular fractionation assays revealed that oxidative stress (H_2_O_2_) significantly increased the level of HDAC4 in the cytosolic fraction, while simultaneously decreasing cytosolic Dnajb5 levels, indicating its translocation to the nucleus (Figure 5f). This suggests that under stress conditions, the nucleocytoplasmic shuttling of these proteins—HDAC4 export and Dnajb5 import—leads to the relief of MEF2 repression, thereby promoting *Ppargc1a* transcription.

### 3.7 Dnajb5 Depletion Improves Muscle Performance by Enhancing Mitochondrial Biogenesis In Vivo

**FIGURE 6:**
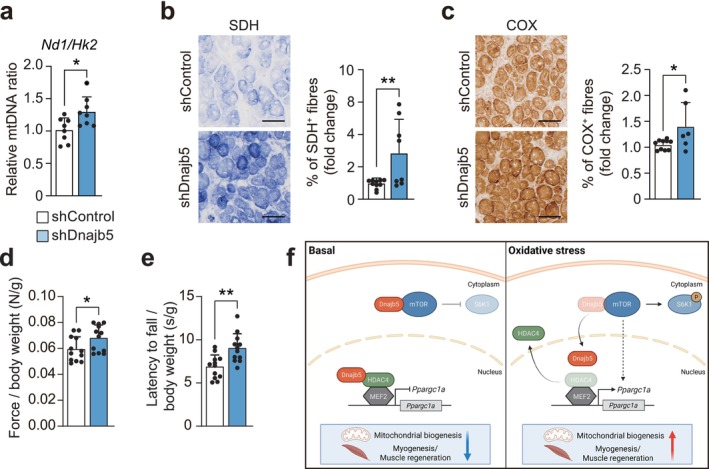
Dnajb5 knockdown enhances mitochondrial biogenesis and muscle performance in vivo. (a) Mitochondrial DNA copy number (*Nd1*/*Hk2* ratio) (expressed as a fold change) in TA muscles 5 days after BaCl_2_ injury and shRNA injection (*n* = 8). (b,c) Representative images and quantification (expressed as a fold change) of (b) SDH (*n* = 8–10) and (c) COX (*n* = 6–10) histochemical staining in regenerating TA muscles. Scale bars: 60 μm. (d) Grip strength normalized to body weight (N/g) (*n* = 12). (e) Rotarod performance (latency to fall) normalized to body weight (s/g) (*n* = 12). (f) Proposed model of Dnajb5‐mediated regulation. Under basal conditions (left), Dnajb5 interacts with mTOR in the cytoplasm to inhibit mTORC1 and binds HDAC4 in the nucleus to repress *Ppargc1a*. Under oxidative stress (right), Dnajb5 translocates to the nucleus but fails to retain HDAC4 due to redox‐dependent dissociation. This loss of interaction facilitates the nuclear export of HDAC4 and the activation of mTORC1, cooperatively promoting myogenesis and mitochondrial biogenesis. Data are presented as mean ± SD. *p < 0.05, **p < 0.01, ***p < 0.001.

To validate the physiological relevance of our mechanistic findings, we assessed mitochondrial function in Dnajb5‐depleted TA muscles in vivo. Consistent with the in vitro data, Dnajb5 knockdown significantly increased mitochondrial biogenesis, as evidenced by a 1.3‐fold increase in the mitochondrial DNA (mtDNA) copy number (*Nd1*/*Hk2* ratio) (Figure 6a). Histochemical staining further demonstrated markedly elevated enzymatic activity in Dnajb5 knockdown muscles, with a 2.8‐fold increase in SDH^+^ fibres and a 1.4‐fold increase in COX^+^ fibres compared with shControl (Figure 6b,c). These mitochondrial improvements translated into measurable performance benefits. Normalized grip strength (force/body weight) was modestly increased (~14%), and rotarod endurance (latency to fall/body weight) was substantially prolonged (~31%) in the Dnajb5 knockdown group (Figure 6d,e). Collectively, our findings demonstrate that Dnajb5 functions as a critical negative regulator of myogenesis and mitochondrial biogenesis through two independent yet complementary pathways (Figure 6f). Under basal conditions, Dnajb5 resides in the nucleus bound to HDAC4, stabilizing the HDAC4‐MEF2 repressor complex to inhibit Ppargc1a expression. Simultaneously, Dnajb5 binds to mTOR in the cytoplasm, restricting mTORC1 signalling. Upon oxidative stress or Dnajb5 depletion, two key events occur: (1) HDAC4 dissociates from the nuclear complex and translocates to the cytosol due to the loss of its retention factor Dnajb5, thereby relieving MEF2 repression and inducing *Ppargc1a*; and (2) Dnajb5 dissociates from mTOR (or is absent), leading to mTORC1 activation. This dual‐release mechanism synergistically enhances protein synthesis and mitochondrial capacity, ultimately improving muscle regeneration and physical performance.

## 4 Discussion

We identified Dnajb5 as a novel mTOR‐binding protein that functions as a negative regulator of muscle differentiation, regeneration and mitochondrial biogenesis. The discovery of this interaction in Antarctic fish muscle, followed by functional validation in mammalian C2C12 myoblasts, primary myoblasts and mouse injury models, demonstrates the evolutionary conservation of this regulatory mechanism and highlights the value of extreme‐environment species for uncovering novel regulators of muscle biology.

As a member of the DnaJ/HSP40 cochaperone family [21], Dnajb5 likely modulates mTOR through direct protein–protein interactions that influence complex stability or enzymatic activity. The critical role of DnaJ proteins in muscle function is exemplified by DNAJB6, where dominant mutations cause limb‐girdle muscular dystrophy type D1 (LGMDD1) through aberrant HSP70 interactions that lead to protein aggregation and muscle dysfunction [22].

Redox regulation is a critical modulator of muscle stem cell activation and differentiation during regeneration [23]. In line with this, our data indicate that oxidative stress significantly reduces the detectable association between Dnajb5 and mTOR, indicating that this interaction is dynamically regulated by redox conditions. While this observation may have implications for pathological states characterized by oxidative stress, including aging, cachexia and inflammatory myopathies [24], further studies will be required to confirm its physiological and pathological significance.

We demonstrated that Dnajb5 functions as a key integrator of protein synthesis and mitochondrial biogenesis. However, our findings indicate a mechanism more complex than mTOR signalling alone. While Dnajb5 depletion significantly upregulated *Ppargc1a* expression, this upregulation was only partially attenuated by rapamycin, suggesting the involvement of an additional, mTOR‐independent pathway. We identified this mechanism as the HDAC4‐MEF2 axis. Consistent with previous structural studies showing that oxidative stress reduces the binding affinity of Dnajb5 for class IIa HDACs [20], our data suggest that the loss of Dnajb5 interaction—whether through depletion or oxidative stress—triggers the nuclear export of HDAC4. Consequently, HDAC4 accumulates in the cytoplasm, thereby relieving the repression of MEF2 and driving *Ppargc1a* transcription. Collectively, these findings indicate that Dnajb5 operates via a ‘dual‐pathway’ mechanism: it restricts anabolism by inhibiting mTORC1 and simultaneously limits mitochondrial biogenesis by maintaining nuclear HDAC4. The coordinated release of these two ‘brakes’ upon Dnajb5 depletion leads to the observed enhancement in both contractile and metabolic capacity, resulting in improved muscle strength and endurance. Notably, our findings demonstrate that the Dnajb5‐mTOR interaction is sensitive to cellular redox states. Since physical exercise is a well‐known physiological inducer of ROS generation in skeletal muscle [25, 26], it is plausible that the Dnajb5‐mediated dual‐pathway identified here may operate during exercise or the post‐exercise recovery phase to coordinate muscle adaptation and mitochondrial biogenesis.

Notably, Dnajb5 knockdown enhanced S6K1 phosphorylation under basal conditions but increased both S6K1 and Akt phosphorylation during myogenic differentiation. This context‐dependent effect on Akt appears to result from mTORC1‐driven IGF2 induction rather than direct mTORC2 regulation. mTOR activation promotes IGF2 production, which subsequently stimulates the IGF1R–PI3K–Akt pathway through autocrine signalling [3]. Consistent with this, Dnajb5 depletion significantly enhanced *Igf2* expression during differentiation. These findings suggest that Dnajb5 acts primarily as a selective negative regulator of mTORC1, with Akt activation during differentiation representing a secondary consequence of IGF2‐mediated signalling.

The temporal regulation of Dnajb5 during muscle regeneration provides important physiological insights, as its natural downregulation following injury suggests that Dnajb5 normally constrains regenerative responses. Notably, although Dnajb5 knockdown did not significantly alter the population of satellite cells (Pax7^+^) or the number of differentiated myogenin^+^ cells, it robustly increased the cross‐sectional area of regenerating myofibres and upregulated myogenic transcripts (*Myog* and *Igf2*). This indicates that Dnajb5 primarily restricts myofibre hypertrophy and transcriptional activation of myogenesis rather than regulating the initial expansion of the satellite cell pool. This suggests that Dnajb5 acts as a molecular brake on the hypertrophic growth and transcriptional priming of regenerating fibres.

The coordinated improvement of muscle differentiation, mitochondrial biogenesis and functional performance following Dnajb5 depletion suggests potential therapeutic relevance for muscle wasting conditions. Targeting Dnajb5 offers advantages over direct mTOR manipulation. Systemic mTOR activation carries risks of metabolic dysfunction, insulin resistance and oncogenic transformation [1, 27]. In contrast, our data suggest that Dnajb5 modulation may promote beneficial muscle adaptations without apparent adverse consequences, although long‐term effects remain to be investigated. The muscle‐enriched expression of Dnajb5 further supports its suitability as a therapeutic target with reduced off‐target effects.

Several limitations warrant consideration. The proteomic analysis was restricted to one Antarctic species, and cross‐species validation would strengthen evolutionary conclusions. The use of knockdown rather than genetic deletion may underestimate the full functional impact of Dnajb5. Additionally, while functional benefits and the involvement of the HDAC4‐MEF2 axis are demonstrated, the precise structural basis of the Dnajb5‐mTOR and Dnajb5‐HDAC4 interactions remains to be characterized. Future research priorities include developing specific Dnajb5 inhibitors, investigating Dnajb5 expression in human muscle disorders, and examining its role in other muscle wasting contexts, such as denervation and disuse atrophy. Long‐term studies assessing compensatory mechanisms and safety of sustained Dnajb5 inhibition are also needed.

In conclusion, Dnajb5 represents a conserved, stress‐responsive regulator that coordinates muscle anabolism and mitochondrial biogenesis through a dual mechanism involving selective mTORC1 inhibition and modulation of the HDAC4‐MEF2 axis. Identified initially in Antarctic fish and functionally validated in mammalian systems, Dnajb5 exemplifies how evolutionary discoveries can illuminate conserved pathways of muscle homeostasis. These findings establish a novel regulatory mechanism with potential therapeutic implications for muscle wasting conditions.

## Conflicts of Interest

The authors declare no conflicts of interest.

## Supporting information


**Table S1:** Proteins whose levels increased reproducibly in mTOR immunoprecipitates from the muscles of *Notothenia coriiceps* compared to those from the muscles of *Takifugu obscurus*.
**Table S2:** The primer sequences used for quantitative real‐time polymerase chain reaction.
**Table S3:** Primer sequences for quantifying mitochondrial copy numbers using real‐time polymerase chain reaction.
**Figure S1: Optimization of mTOR immunoprecipitation from fish muscle lysates.** (a) Immunoprecipitation (IP) was performed from fish muscle using different concentrations (2, 4, and 6 μg) of anti‐Raptor antibody to optimize the conditions. (b) IP with anti‐mTOR antibody at different concentrations (2, 4, and 6 μg) showing dose‐dependent isolation of mTOR. (c) Western blot analysis confirming the effective isolation of mTOR complexes, indicated by the co‐immunoprecipitation of Raptor, from *Notothenia coriiceps* muscle using 4μg of anti‐mTOR antibody. (d) Immunoprecipitation with anti‐mTOR antibody using muscle lysates from *N. coriiceps* (NC) and *T. obscurus* (TO). Western blot analysis of mTOR and Raptor confirms the conservation of the mTOR complex in both fish species.
**Figure S2: Identification of Dnajb5 as an mTOR‐binding protein by LC–MS/MS and RT‐PCR.** (a) Total ion chromatogram of mTOR immunoprecipitates from in‐gel‐digested *Notothenia coriiceps* (NC). (b) MS/MS spectrum of the specific peptide for Dnajb5. Annotated sequence: [R]SHSRSNGFSFHNDHDAEQDMDMEEEDPFAHIGR[Q]. (c) RT‐PCR analysis showing expression of *mtor* and *dnajb5* in NC muscle.
**Figure S3: Dnajb5 mRNA expression during myogenesis and its interaction with mTOR in HEK293 cells.** (a) Relative mRNA expression (expressed as a fold change) of *Dnajb5* in C2C12 cells at day 0 (0D) and day 2 (2D) of differentiation. The graph shows the transcriptional upregulation of Dnajb5 upon differentiation. Data are presented as mean ± SD; ***P < 0.001. (b) Co‐immunoprecipitation (Co‐IP) of Flag‐tagged Dnajb5 with endogenous mTOR in HEK293 cells. HEK293 cells were transfected with a Flag‐Dnajb5 expression vector. Lysates were immunoprecipitated (IP) with an anti‐Flag antibody, followed by western blot analysis of mTOR and Flag‐Dnajb5. Tubulin was used as a loading control for the whole cell lysates (Input).
**Figure S4: Dnajb5 knockdown does not affect FBS‐induced mTOR activation.** C2C12 cells were transduced with shControl or Dnajb5 shRNAs (shDnajb5 #1 and #2). After 5 days of puromycin selection (3 μg/mL), cells were serum‐starved for 18 h and stimulated with 10% FBS. Cell lysates were subjected to western blot analysis of Dnajb5, Akt, p‐Akt (S473), S6K1, and p‐S6K1 (T389). Tubulin served as a loading control. Quantification of band intensities (expressed as a fold change) is shown below. Note that while Dnajb5 protein levels were significantly reduced, the phosphorylation levels of Akt and S6K1 remained unchanged. Data are presented as mean ± SD (n = 3). Statistical significance was determined by an unpaired *t* test. *P < 0.05, **P < 0.01, ***P < 0.001.
**Figure S5: Dnajb5 knockdown does not alter the total number of regenerating myofibers.** Quantification of the total number of regenerating myofibers in the injured tibialis anterior (TA) muscle sections at 5 days post‐injury. Note that while the fiber cross‐sectional area (CSA) was increased (as shown in Figure 4e), the total number of fibers remained unchanged between shControl and shDnajb5 groups. Data are presented as mean ± SD. Individual data points represent biological replicates (n = 7–8). No statistically significant difference was observed (unpaired *t* test).
**Figure S6: Dnajb5 knockdown enhances Ppargc1a expression.** Relative mRNA expression (expressed as a fold change) of *Dnajb5* and *Ppargc1a* in C2C12 myoblasts at day 0 (0D) and day 2 (2D) of differentiation following shControl or shDnajb5 knockdown (shDnajb5 #1 and #2). Note that *Ppargc1a* levels are significantly increased in Dnajb5‐depleted cells. Data are presented as mean ± SD. Individual data points represent biological replicates (n = 3). Statistical significance was determined by an unpaired *t* test (two groups). *P < 0.05, **P < 0.01, ***P < 0.001.
