# Supplementary material for: Dnajb5 From Antarctic Fish Reveals a Redox‐Sensitive Mechanism Coordinating Muscle Regeneration via mTORC1 and HDAC4
Source: J Cachexia Sarcopenia Muscle. 2026 Jul 10;17(4):e70332. doi: 10.1002/jcsm.70332 (PMC13355398; doi:10.1002/jcsm.70332)
Supplement: Supplementary file 1 — Table S1: Proteins whose levels increased reproducibly in mTOR immunoprecipitates from the muscles of Notothenia coriiceps compared to those from the muscles of Takifugu obscurus. Table S2: The primer sequences used for quantitative real‐time polymerase chain reaction. Table S3: Primer sequences for quantifying mitochondrial copy numbers using real‐time polymerase chain reaction. Figure S1: Optimization of mTOR immunoprecipitation from fish muscle lysates. (a) Immunoprecipitation (IP) was performed from fish muscle using different concentrations (2, 4, and 6 μg) of anti‐Raptor antibody to optimize the conditions. (b) IP with anti‐mTOR antibody at different concentrations (2, 4, and 6 μg) showing dose‐dependent isolation of mTOR. (c) Western blot analysis confirming the effective isolation of mTOR complexes, indicated by the co‐immunoprecipitation of Raptor, from Notothenia coriiceps muscle using 4μg of anti‐mTOR antibody. (d) Immunoprecipitation with anti‐mTOR antibody using muscle lysates from N. coriiceps (NC) and T. obscurus (TO). Western blot analysis of mTOR and Raptor confirms the conservation of the mTOR complex in both fish species. Figure S2: Identification of Dnajb5 as an mTOR‐binding protein by LC–MS/MS and RT‐PCR. (a) Total ion chromatogram of mTOR immunoprecipitates from in‐gel‐digested Notothenia coriiceps (NC). (b) MS/MS spectrum of the specific peptide for Dnajb5. Annotated sequence: [R]SHSRSNGFSFHNDHDAEQDMDMEEEDPFAHIGR[Q]. (c) RT‐PCR analysis showing expression of mtor and dnajb5 in NC muscle. Figure S3: Dnajb5 mRNA expression during myogenesis and its interaction with mTOR in HEK293 cells. (a) Relative mRNA expression (expressed as a fold change) of Dnajb5 in C2C12 cells at day 0 (0D) and day 2 (2D) of differentiation. The graph shows the transcriptional upregulation of Dnajb5 upon differentiation. Data are presented as mean ± SD; ***P < 0.001. (b) Co‐immunoprecipitation (Co‐IP) of Flag‐tagged Dnajb5 with endogenous mTOR in HEK293 cells. HEK293 cel [file JCSM-17-e70332-s001.docx]

**Dnajb5 from Antarctic fish reveals a redox-sensitive mechanism coordinating muscle regeneration via mTORC1 and HDAC4**

Sun-Hee Cho^1#^, Min-Kyung Choi^2#^, Mi-Ock Baek^2,3^, Hyeon-Hee Son^4^, Binh Thanh Nguyen^5, 6^, Min-Jung Kang^5, 6^, Il-Chan Kim^7^, Jin-Hyoung Kim^7, 8^, Mee-Sup Yoon^1, 2, 3, 4*^

***** Correspondence to: Mee-Sup Yoon, E-Mail: [msyoon@gachon.ac.kr](mailto:msyoon@gachon.ac.kr),

**Supplementary information includes:**

Supplementary Methods and Materials
Table S1–S3

Figure S1–S6

**Supplementary Methods and Materials**

***Reagents and antibodies***

The following antibodies were used in the study: anti-Akt (#9272), anti-pS473-Akt (#4051), anti-S6K1 (#9202), anti-pThr389-S6K1 (#9234), anti-HDAC4 (#7628) were obtained from Cell Signaling Technology (Danvers, MA, USA). Anti-Tubulin (ab11304) and anti-Ki67 (ab15580) were purchased from Abcam (Cambridge, UK). Antibodies against MHC (MF20-C), myogenin (F5D), and Pax7 (Pax7-C) were obtained from Developmental Studies Hybridoma Bank (DSHB), created by the NICHD of the NIH and maintained at The University of Iowa (Iowa City, IA, USA). Anti-Dnajb5 (PA1-776) was obtained from Invitrogen (Waltham, MA, USA) and anti-laminin (L9393) was from Sigma-Aldrich (St. Louis, MO, USA). Anti-Raptor (A300-553A) was obtained from Bethyl Laboratories (Montgomery, TX, USA). Antibodies against mTOR (sc-517464) and Lamin B (sc-374015) were obtained from Santa Cruz Biotechnology (Santa Cruz, CA, USA). Horseradish peroxidase (HRP)-conjugated anti-mouse (115-035-003) and anti-rabbit (211-002-171) were purchased from Jackson ImmunoResearch Laboratories (West Grove, PA, USA). Chemical reagents including rapamycin and hydrogen peroxide (H_2_O_2_) were purchased from Sigma-Aldrich, and Trichostatin A (TSA) was obtained from Selleckchem (Houston, TX, USA).

**Immunoprecipitation analysis in fish skeletal muscles and HEK 293 cells**

*Fish muscle lysis:* Dorsal skeletal muscle from *Notothenia* *coriiceps* and *Takifugu* *obscurus* was snap-frozen in liquid nitrogen and stored at −80 °C. The tissues (30−50 mg) were homogenized using 5 mm steel beads and a TissueLyser II (Qiagen, Hilden, Germany) at 50 Hz for 3 min in a lysis buffer containing 40 mM HEPES buffer (pH 7.4), 120 mM NaCl, 10 mM sodium pyrophosphate, 50 mM NaF, 2 mM EDTA, and 0.3% CHAPS supplemented with protease inhibitor cocktail (#04693159001, Roche, Basel, Switzerland) and phosphatase inhibitors (Roche, #04906837001). Lysates were centrifuged at 13,000 × g for 10 min at 4°C, and the supernatants were collected for further analysis.

***Antibody validation and Immunoprecipitation (fish):* To confirm** antibody cross-reactivity and optimize conditions for *N. coriiceps* and *T. obscurus*, we first performed small-scale IP using commercially available anti-mTOR and anti-Raptor antibodies by titrating the antibody amount (2, 4, and 6 µg) with a fixed lysate input (1.0 mg per reaction). Species-matched normal IgG served as a negative control. Following validation of antibody specificity, large-scale immunoprecipitation was performed by incubating the lysates with anti-mTOR antibodies at 4°C overnight. The immune complexes were then captured by incubating with protein G agarose beads (Millipore, Molsheim, France) for 1 h at 4°C, followed by three washes with the lysis buffer to remove non-specifically bound proteins.

***In-gel digestion and sample clean-up***

After immunoprecipitation with mTOR antibodies in muscle cells of both *N. coriiceps* and *T. obscurus*, mTOR-binding proteins were separated by SDS-PAGE gel and stained with a silver stain kit (Sigma-Aldrich, St. Louis, MO, USA). The resulting band was then cut into 1 to 2 mm pieces for reduction (1 mM DTT, 60°C, 1 h) and alkylation (55 mM iodoacetamide, room temperature, in the dark). Following dehydration with 100% acetonitrile, the gel pieces were rehydrated in 100 μL of 15 ng/ μL trypsin solution and 50 mM ammonium hydrogen carbonate buffer. Trypsin was added at a 1:30 ratio (trypsin: protein, w/w) and incubated at 37°C for 24 h. After incubation, peptides were extracted using a 1:2 (v/v) mixed solvent of 5% formic acid/acetonitrile and concentrated by centrifugation under reduced pressure using an Alpha 1-2 LD plus vacuum freeze dryer (Martin Christ, Niedersachsen, Germany). The samples were cleaned up using a C18 spin column (Pierce, MA, USA) following the manufacturer's guidelines and stored at −20°C until mass spectrometry analysis.

***Nano liquid chromatography-LTQ Orbitrap analysis***

Samples were reconstituted with 50 μL of 0.1% formic acid and analyzed using an LTQ Orbitrap Velos Pro mass spectrometer (Thermo Fisher Scientific, Sunnyvale, CA, USA) connected with an EASY-nLC1000 liquid chromatography system. Distilled water containing 0.1% formic acid (solvent A) and acetonitrile containing 0.1% formic acid (solvent B) were used as solvents. The nano liquid chromatography system used a column constructed by connecting 2 cm of a C18 trap nanocolumn (Acclaim PepMap 100, 75 μm; Thermo Scientific) to 50 cm of an Easy-Spray column (EASY-Spray column, PepMap, 75 μm; Thermo Scientific). A solvent mixture containing solvents A and B was run for 120 min at a flow rate of 300 nL/minute with a gradient of 5 to 40% of solvent B. A spray voltage of 1.8 kV and an ion transfer tube temperature of 275°C were used in positive mode. A maximum injection volume of 3 μL was used during data acquisition in partial injection mode. The collision-induced dissociation (CID) energy was set at 35 V, with variables including an exclusion time of 180 seconds, 2-second repetitions, a repetition period of 30 seconds, an exclusion mass width of 10 ppm, and an exclusion size of 500. Only singly charged ions were excluded from the collection. Muscle samples were analyzed in three separate sets. The mass spectrometer was operated in a data-dependent mode, automatically switching between MS and MS/MS acquisition. MS/MS data acquisition and processing were done using Xcalibur™ software (Thermo Scientific, MA, USA).

***Identification of proteins***

Proteins were identified from the peaks detected using the nano-liquid chromatography-LTQ Orbitrap mass spectrometer with Proteome Discover ver. 2.5 (Thermo Fisher Scientific, Waltham, MA, USA). The protein sequences of *N. coriiceps* and *T. obscurus* were obtained from the UniProt protein database. In SEQUEST searches for standard peptides, carbamidomethylation of cysteine was used as a static modification, and methionine oxidation as a dynamic modification. Trypsin was selected as the proteolytic enzyme with allowance for two missed cleavages. Peptide and fragment mass tolerances were set at ± 1.6 and 0.6 Da, with a precursor mass range of 350–5000 Da, and peptide charges were set to exclude +1. SEQUEST HT results were filtered using Percolator-based scoring to improve the sensitivity and accuracy of peptide identification. Statistical analysis between different groups was conducted using a chi-square test. The proteomic data set was used to calculate the fold change between *N. coriiceps* and *T. obscurus*.

***Plasmid construction and Immunoprecipitation in HEK293 cells***

*Plasmid construction*: The coding sequence of DNAJB5 was amplified by PCR using the pcDNA5/FRT/TO-GFP-DNAJB5 plasmid (Addgene plasmid #19500) as a template. The PCR product was cloned into the BamHI/XhoI sites of the pcDNA-Flag-UNE-L [1] using the EZ-Fusion™ HT Cloning Kit (Enzyomics, Daejeon, Korea). The resulting construct, pcDNA-Flag-Dnajb5, encodes N-terminally Flag-tagged Dnajb5.

*Immunoprecipitation (Flag-IP):* For validation of the interaction in a heterologous system, HEK293 cells were transfected with the Flag-tagged Dnajb5 constructs described above. Cells were lysed in the same lysis buffer used for C2C12 cells. The lysates were incubated with anti-Flag M2 affinity gels (A2220, Sigma-Aldrich, Burlington, MA, USA) overnight at 4°C. The immunoprecipitates were washed three times with lysis buffer, eluted by boiling in SDS sample buffer, and analyzed by western blotting.

***RNA isolation and quantitative real-time polymerase chain reaction (qRT-PCR)***

Total RNA was isolated from skeletal muscle tissues (about 30 mg) and C2C12 cells using TRIzol reagent (Thermo Fisher Scientific, Waltham, MA, USA). Muscle tissue samples were homogenized using steel beads and a tissue disruption machine (TissueLyser II, Qiagen) set at an oscillation frequency of 30 Hz for 1-2 min [2]. Complementary DNA (cDNA) was generated using the TOPscriptTM RT DryMIX kit (dT18 plus) from Enzynomics. Quantitative real-time polymerase chain reaction (qPCR) analysis was performed using the TOPrealTM qPCR 2× PreMIX (SYBR Green with high ROX) (Enzynomics) and a CFX384 C1000 Thermal Cycler (Bio-Rad, Hercules, CA, USA) [3, 4]. Relative gene expression levels were calculated using the comparative C_t_ method (2 ^−ΔΔCt^). Glyceraldehyde-3-phosphate dehydrogenase (*Gapdh*) was used as a housekeeping reference gene. Primer sequences used in the study are listed in Table S2.

***Mitochondrial DNA copy number***

The mitochondrial DNA (mtDNA) copy number was evaluated using methods outlined in Quiros et al. [5]. The relative mtDNA copy number was determined by calculating the ratio of mtDNA to nuclear DNA (nDNA) based on the ΔCt values of the mitochondrial gene NADH dehydrogenase subunit 1 (*Nd1*) and the nuclear reference gene hexokinase 2 (*Hk2*). The primer sequences are listed in Table S3.

**Table S1.** Proteins whose levels increased reproducibly in mTOR immunoprecipitates from the muscles of *Notothenia coriiceps* compared to those from the muscles of *Takifugu obscurus.*

| **No.** | **Protein FDR Confidence** | **Accession** | **Description** | **Coverage [%]** | **MW [kDa]** | **Abundance Ratio** | **Reproducibility** |
| --- | --- | --- | --- | --- | --- | --- | --- |
| 1 | Low | XP_010764355.1 | ubiquitin-like protein 5 | 11 | 8.5 | 100 | 6/9 |
| 2 | Low | XP_010765141.1 | alpha-tocopherol transfer protein, partial | 11 | 26.7 | 100 | 6/9 |
| **3** | **High** | **XP_010765758.1** | **heterogeneous nuclear ribonucleoprotein C-like** | **27** | **9.1** | **80.2** | **9/9** |
| 4 | Low | XP_010768261.1 | uncharacterized protein C7orf62 homolog | 4 | 28.7 | 100 | 6/9 |
| 5 | High | XP_010771894.1 | serine/threonine-protein kinase mTOR-like, partial | 13 | 35.2 | 100 | 6/9 |
| **6** | **High** | **XP_010772209.1** | **dnaJ homolog subfamily B member 5** | **9** | **41.4** | **100** | **6/9** |
| 7 | Low | XP_010773864.1 | potassium/sodium hyperpolarization-activated cyclic nucleotide-gated channel 4-like | 3 | 168.4 | 66.9 | 6/9 |
| 8 | Low | XP_010775519.1 | paraspeckle component 1-like | 36 | 18.7 | 100 | 6/9 |
| 9 | Low (exclusive) | XP_010778821.1 | supervillin-like isoform X1 | 1 | 169.9 | 86.1 | 6/9 |
| 10 | Low | XP_010787404.1 | calcium permeable stress-gated cation channel 1-like | 26 | 20.4 | 100 | 6/9 |
| 11 | Low | XP_010792598.1 | transcription factor E4F1-like | 2 | 58.3 | 100 | 6/9 |
| 12 | Low | JAO79375.1 | NAL12, partial | 2 | 42.7 | 100 | 4/9 |
| **13** | **High** | **JAO73270.1** | **GELS, partial** | **3** | **40.3** | **100** | **4/9** |
| 14 | Low | JAO39712.1 | RFXK | 5 | 18.4 | 100 | 4/9 |
| 15 | Low | XP_010767018.1 | PREDICTED: 5-hydroxytryptamine receptor 3A-like | 9 | 27.2 | 100 | 4/9 |
| 16 | Low | JAO48533.1 | LIN54, partial | 9 | 35.8 | 100 | 4/9 |
| 17 | Low | JAO55392.1 | CRGM2, partial | 16 | 22.6 | 100 | 4/9 |
| 18 | Low | XP_010767023.1 | PREDICTED: UPF0687 protein C20orf27 homolog | 25 | 19 | 100 | 4/9 |
| 19 | Low | XP_010781815.1 | PREDICTED: protein FAM161A-like isoform X2 | 3.6 | 38.7 | 100 | 4/9 |
| 20 | Low | XP_010793403.1 | PREDICTED: ras-like protein family member 10B isoform X1 | 4.4 | 23.2 | 100 | 4/9 |
| 21 | Low | JAO51735.1 | TNNI2 | 4.8 | 18.9 | 100 | 4/9 |

coverage > 2%, reproducibility ≥ 4/9, abundance ratio > 2

**Table S2.** The primer sequences used for quantitative real-time polymerase chain reaction (qPCR) expression analysis.

| Species | Gene | Forward (5′–3′) | Reverse (5′–3′) |
| --- | --- | --- | --- |
| *Notothenia coriiceps* | *dnajb5* | GGAAAACCGCATCATCTCGC | TGTCGGGAAAACGGACAGAG |
|  | *gapdh* | TCCGGTAAACTGTGGAGGGA | AGCCATGCCGGTGATCTTAC |
|  | *mtor* | AAGAGCAGAGCGCTCGTATG | CTTCCTCATCTCCAGACCGC |
| Mouse | *Ckm* | CACCTCCACAGCACAGACAG | ACCTTGGCCATGTGATTGTT |
|  | *Dnajb5* | GGCACCAACGTGCTCTACAG | ACGGTGCCTGGCTTGATAAC |
|  | *Gapdh* | TCCCACTCTTCCACCTTCGA | CAGGAAATGAGCTTGACAAAGTTG |
|  | *Igf2* | CGCTTCAGTTTGTCTGTTCG | AGGTAGACACGTCCCTCTCG |
|  | *Myog* | TACGTCCATCGTGGACAGCAT | TCAGCTAAATTCCCTCGCTGG |
|  | *Ppargc1a* | CGGAAATCATATCCAACCAG | TGAGGACCGCTAGCAAGTTTG |

Gene abbreviations: *Ckm* (mouse), creatine kinase muscle; *dnajb5* (*N. coriiceps*), DnaJ homolog subfamily member B5; *Dnajb5* (mouse), DnaJ homolog subfamily member B5; *Gapdh* (mouse and *N. coriiceps*), glyceraldehyde-3-phosphate dehydrogenase; *Igf2* (mouse), insulin-like growth factor 2; *mtor* (*N. coriiceps*), serine/threonine-protein kinase mTOR-like; *Myog* (mouse), myogenin; *Ppargc1a* (mouse), peroxisome proliferative activated receptor gamma coactivator 1 alpha.

**Table S3.** Primer sequences for quantifying mitochondrial copy numbers using real-time polymerase chain reaction.

| **Gene** | **Forward sequence (5′–3′)** | **Reverse sequence (5′–3′)** |
| --- | --- | --- |
| *Hk2* | GCCAGCCTCTCCTGATTTTAGTGT | GGGAACACAAAAGACCTCTTCCTGG |
| *Nd1* | CTAGCAGAAACAAACCGGGC | CCGGCTGCGTATTCTACGTT |

Gene abbreviations: *Hk2*: hexokinase 2; *Nd1*: NADH dehydrogenase subunit 1

**
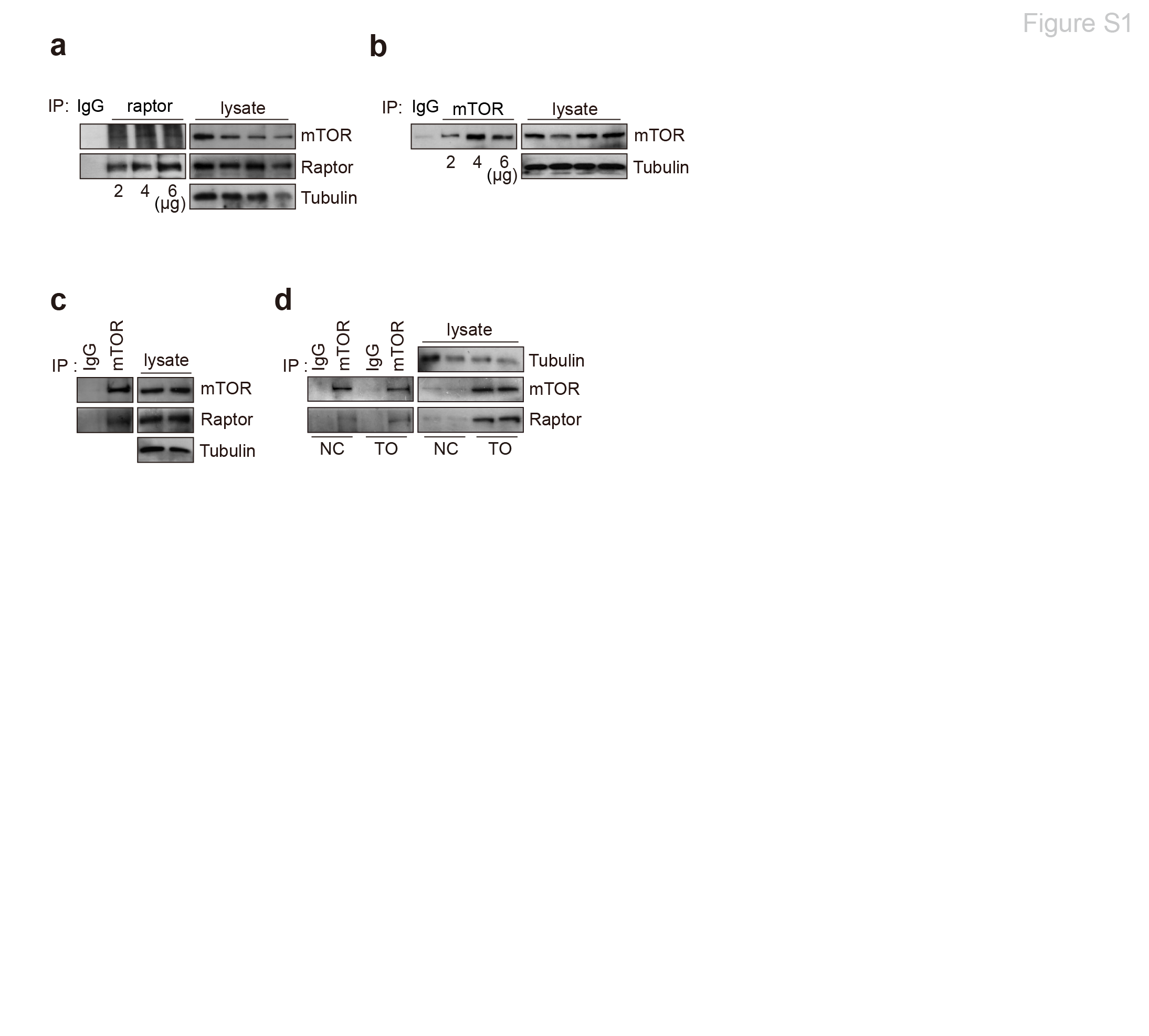
**

**Figure S1. Optimization of mTOR immunoprecipitation from fish muscle lysates.**

(a) Immunoprecipitation (IP) was performed from fish muscle using different concentrations (2, 4, and 6 μg) of anti-Raptor antibody to optimize the conditions. (b) IP with anti-mTOR antibody at different concentrations (2, 4, and 6 μg) showing dose-dependent isolation of mTOR. (c) Western blot analysis confirming the effective isolation of mTOR complexes, indicated by the co-immunoprecipitation of Raptor, from *Notothenia coriiceps* muscle using 4μg of anti-mTOR antibody. (d) Immunoprecipitation with anti-mTOR antibody using muscle lysates from *N. coriiceps* (NC) and *T. obscurus* (TO). Western blot analysis of mTOR and Raptor confirms the conservation of the mTOR complex in both fish species.

**
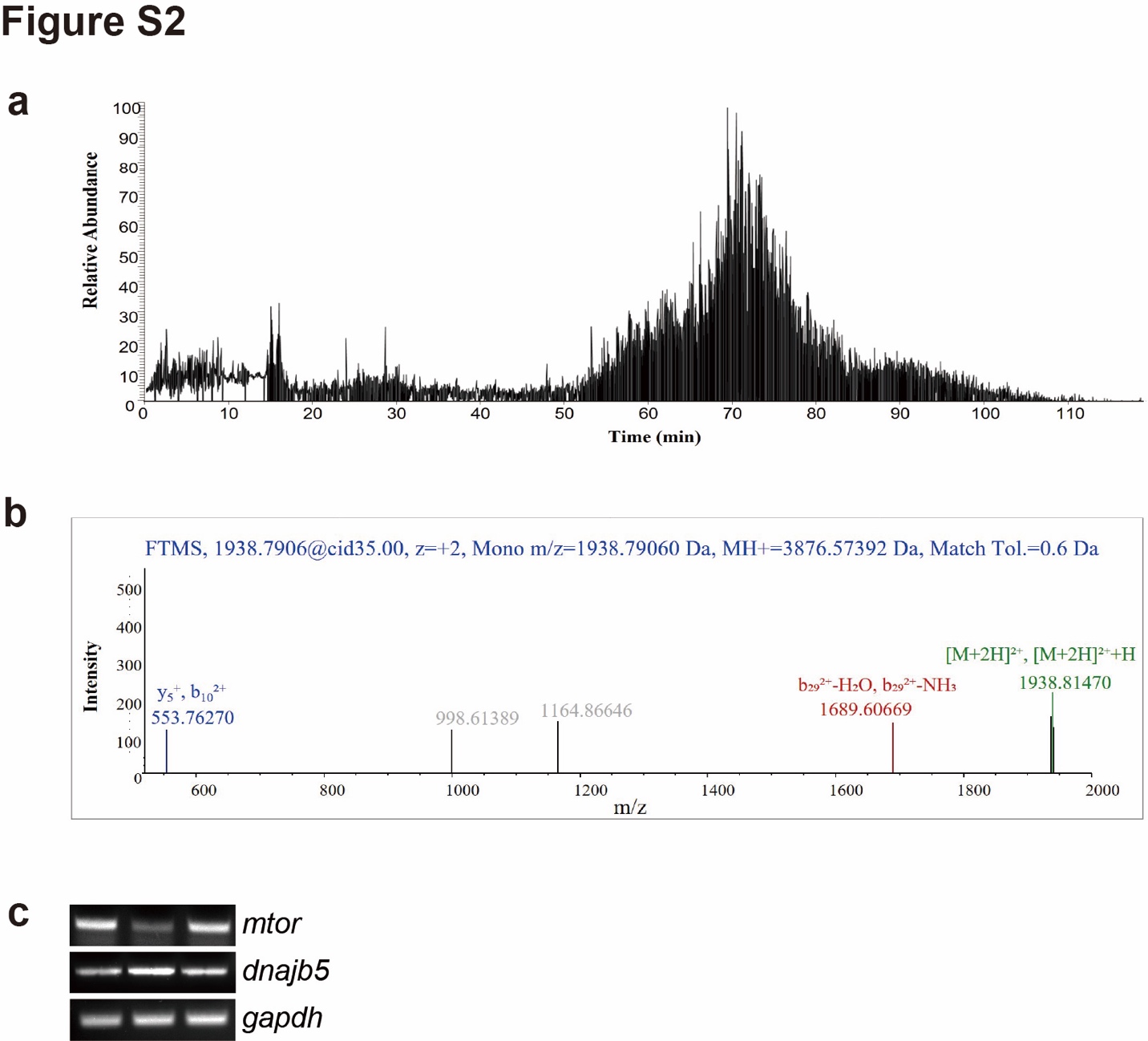
**

**Figure S2. Identification of Dnajb5 as an mTOR-binding protein by LC–MS/MS and RT-PCR.**

(a) Total ion chromatogram of mTOR immunoprecipitates from in-gel-digested *Notothenia coriiceps* (NC). (b) MS/MS spectrum of the specific peptide for Dnajb5. Annotated sequence: [R]SHSRSNGFSFHNDHDAEQDMDMEEEDPFAHIGR[Q]. (c) RT-PCR analysis showing expression of *mtor* and *dnajb5* in NC muscle.

**
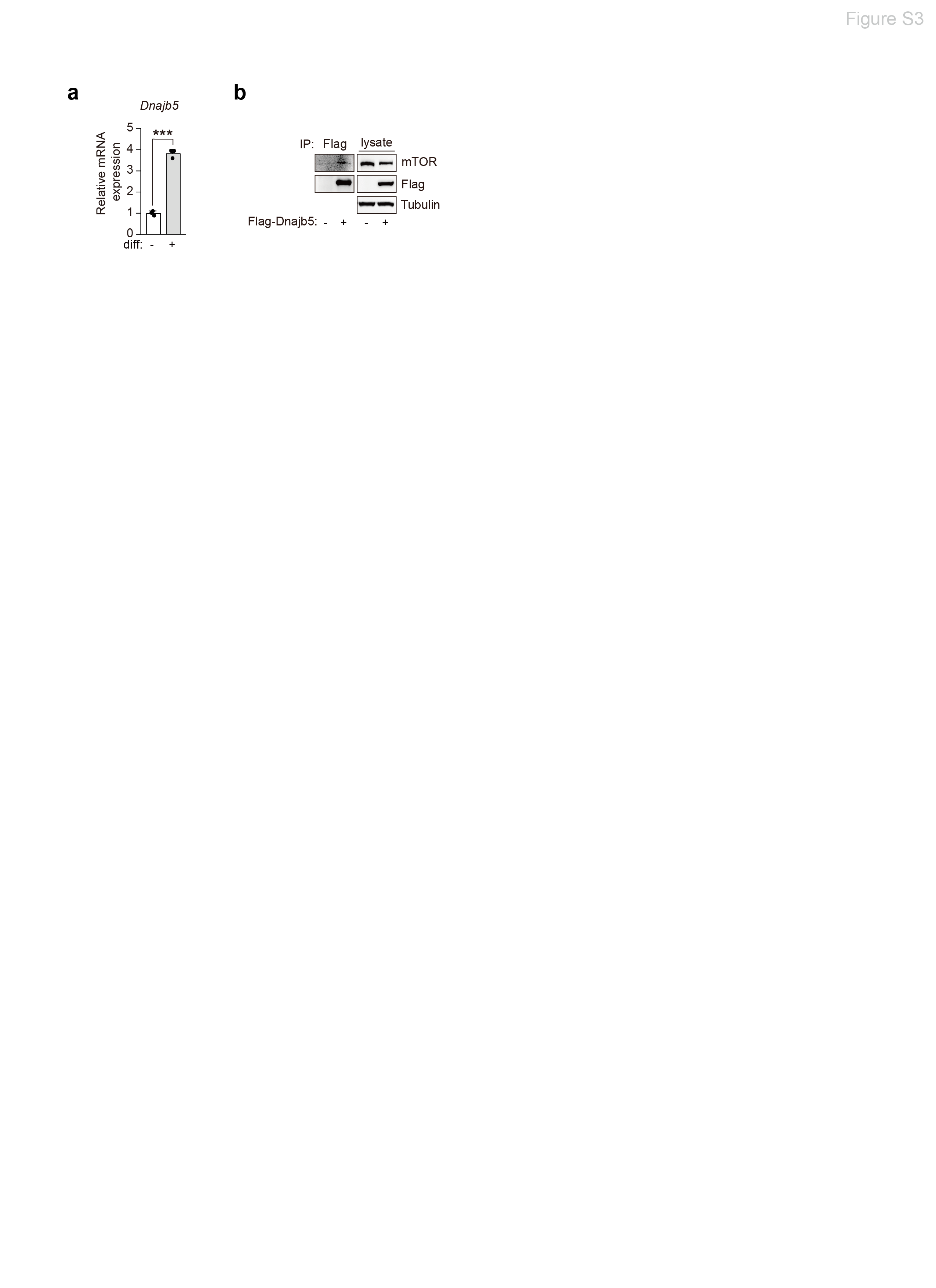
**

**Figure S3. Dnajb5 mRNA expression during myogenesis and its interaction with mTOR in HEK293 cells.**

(a) Relative mRNA expression (expressed as a fold change) of *Dnajb5* in C2C12 cells at day 0 (0D) and day 2 (2D) of differentiation. The graph shows the transcriptional upregulation of Dnajb5 upon differentiation. Data are presented as mean ± SD; ****p* < 0.001. (b) Co-immunoprecipitation (Co-IP) of Flag-tagged Dnajb5 with endogenous mTOR in HEK293 cells. HEK293 cells were transfected with a Flag-Dnajb5 expression vector. Lysates were immunoprecipitated (IP) with an anti-Flag antibody, followed by western blot analysis of mTOR and Flag-Dnajb5. Tubulin was used as a loading control for the whole cell lysates (Input).

**
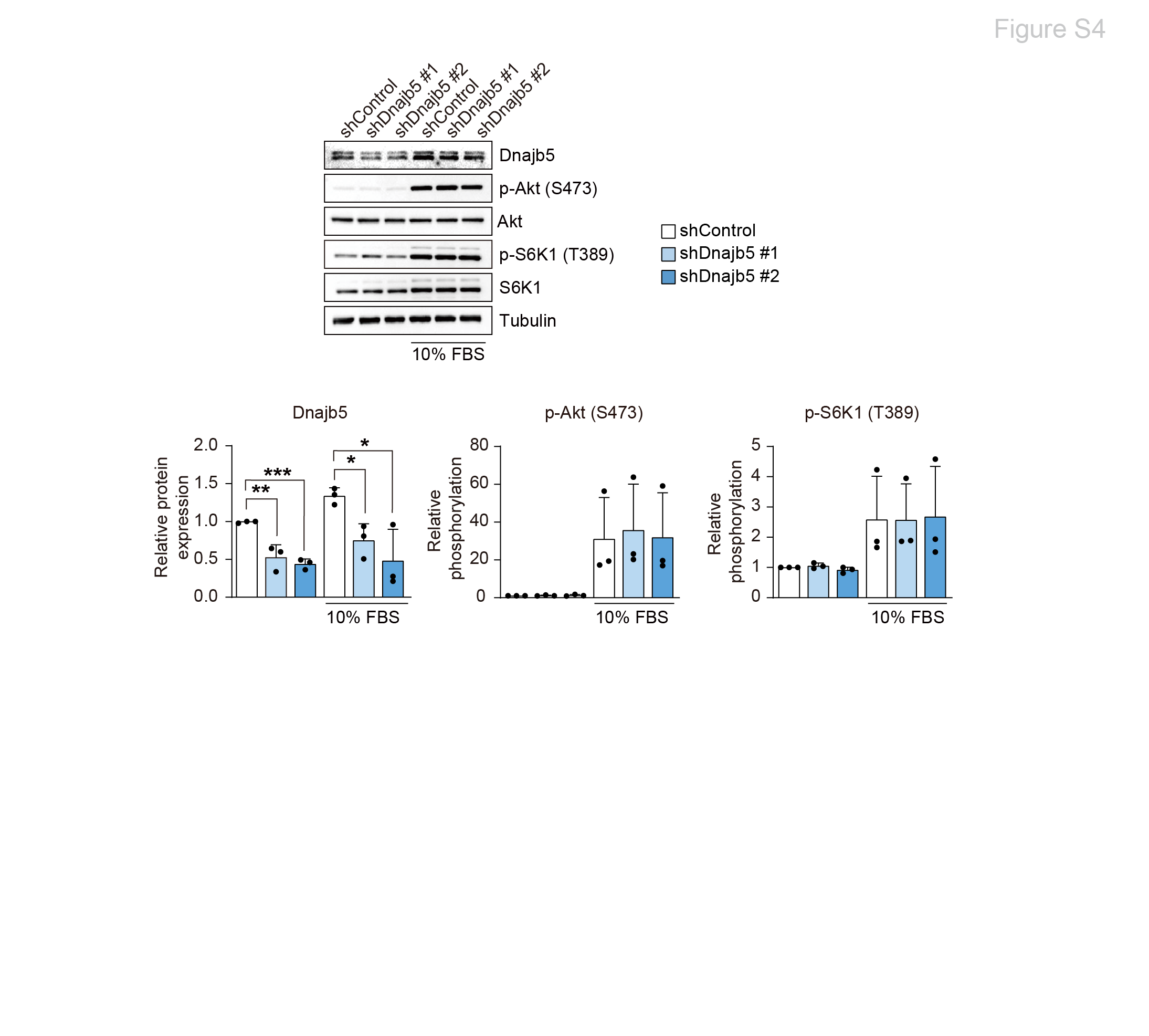
**

**Figure S4. Dnajb5 knockdown does not affect FBS-induced mTOR activation.**

C2C12 cells were transduced with shControl or Dnajb5 shRNAs (shDnajb5 #1 and #2). After 5 days of puromycin selection (3 μg/mL), cells were serum-starved for 18 h and stimulated with 10% FBS. Cell lysates were subjected to western blot analysis of Dnajb5, Akt, p-Akt (S473), S6K1, and p-S6K1 (T389). Tubulin served as a loading control. Quantification of band intensities (expressed as a fold change) is shown below. Note that while Dnajb5 protein levels were significantly reduced, the phosphorylation levels of Akt and S6K1 remained unchanged. Data are presented as mean ± SD (n = 3). Statistical significance was determined by an unpaired *t* test. **p* < 0.05, ***p* < 0.01, ****p* < 0.001.


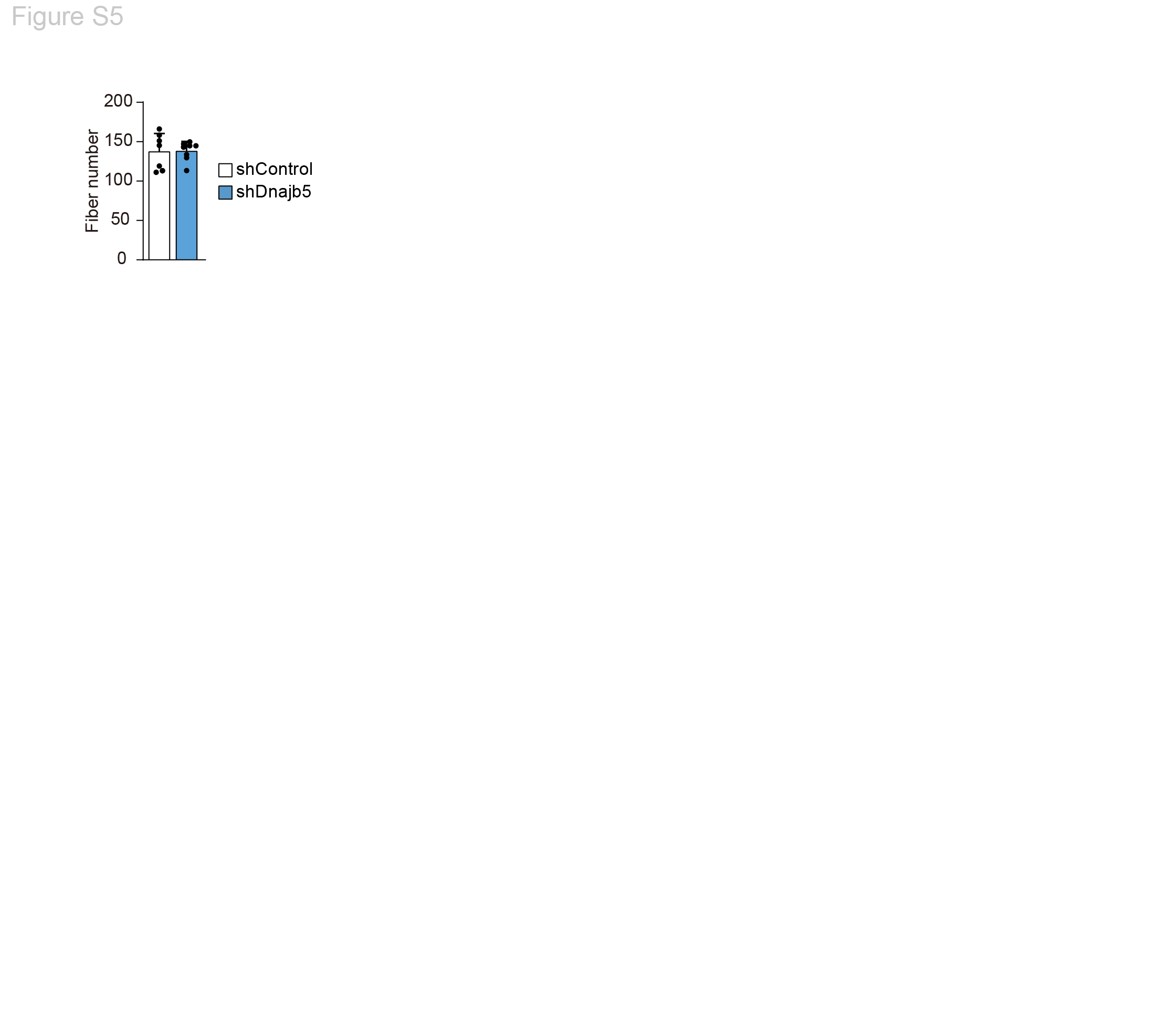


**Figure S5. Dnajb5 knockdown does not alter the total number of regenerating myofibers.** Quantification of the total number of regenerating myofibers in the injured tibialis anterior (TA) muscle sections at 5 days post-injury. Note that while the fiber cross-sectional area (CSA) was increased (as shown in Figure 4e), the total number of fibers remained unchanged between shControl and shDnajb5 groups. Data are presented as mean ± SD. Individual data points represent biological replicates (n = 7–8). No statistically significant difference was observed (unpaired *t* test).

**
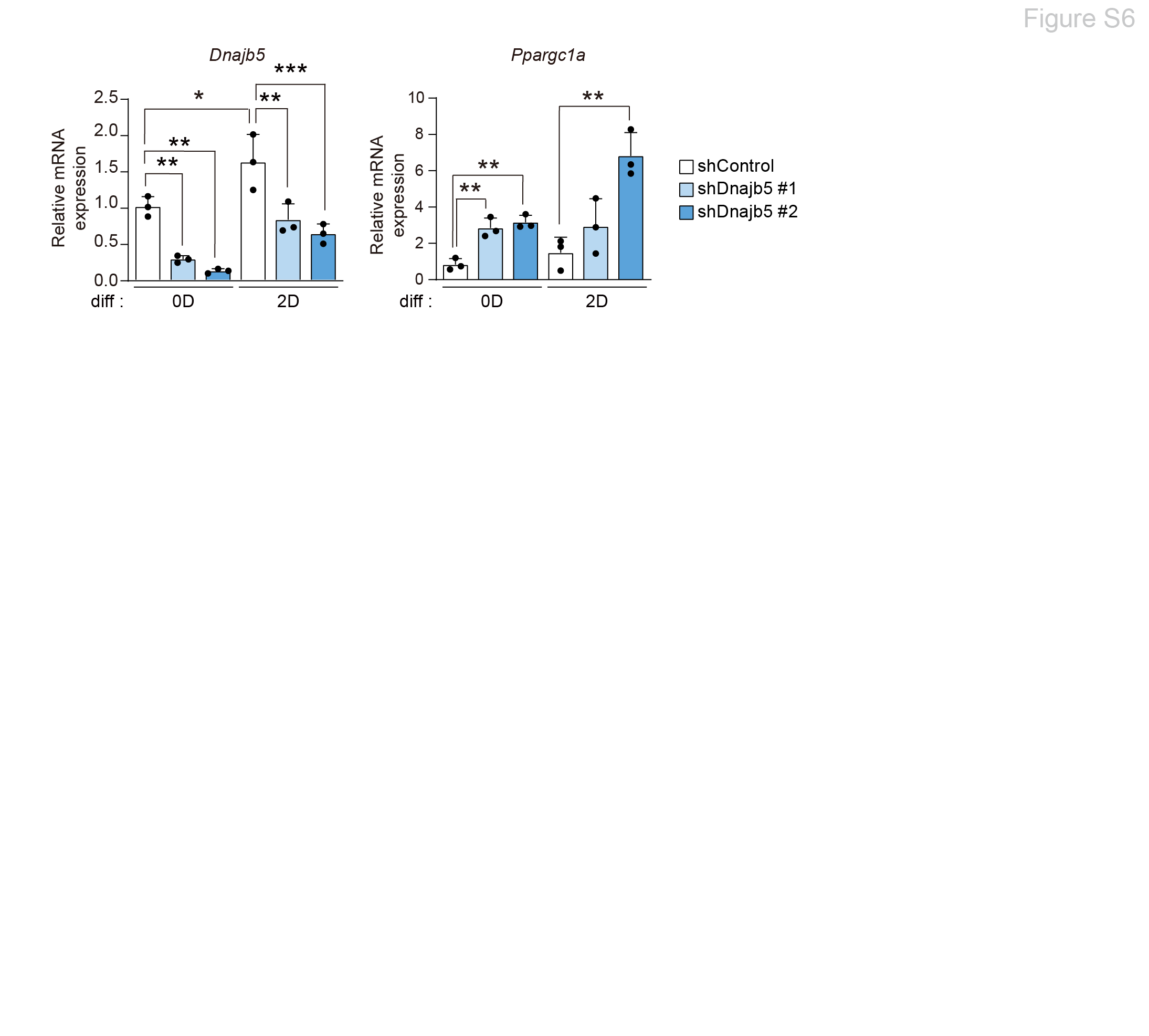
**

**Figure S6. Dnajb5 knockdown enhances *Ppargc1a* expression.**

Relative mRNA expression (expressed as a fold change) of *Dnajb5* and *Ppargc1a* in C2C12 myoblasts at day 0 (0D) and day 2 (2D) of differentiation following shControl or shDnajb5 knockdown (shDnajb5 #1 and #2). Note that *Ppargc1a* levels are significantly increased in Dnajb5-depleted cells. Data are presented as mean ± SD. Individual data points represent biological replicates (n = 3). Statistical significance was determined by an unpaired *t* test (two groups). **p* < 0.05, ***p* < 0.01, ****p* < 0.001.

**Reference**

1 Baek MO, Cho HJ, Min DS, Choi CS, Yoon MS: Self-transducible LRS-UNE-L peptide enhances muscle regeneration. J Cachexia Sarcopenia Muscle 2022;13:1277-1288.

2 Cho SH, Choi B, Lee J, Lee YS, Baek MO, Lee YJ, Gil CO, Choi MK, Khaliq SA, Maham S, Hyun JK, Roh G, Choi H, Lee S, Bae SH, Lee S, Park HJ, Ahn JH, Lee NY, Kang BC, Seo YK, Lim BK, Nam JH, Rho M, Yoon MS: ApoE deficiency protects from mRNA vaccine-induced mitochondrial dysfunction at the injection site under metabolic stress. Theranostics 2025;15:8964-8984.

3 Kim HY, Kim YM, Hong S: DNAJB9 suppresses the metastasis of triple-negative breast cancer by promoting FBXO45-mediated degradation of ZEB1. Cell Death Dis 2021;12:461.

4 Byun KA, Oh S, Batsukh S, Kim MJ, Lee JH, Park HJ, Chung MS, Son KH, Byun K: The Extracellular Matrix Vitalizer RA(TM) Increased Skin Elasticity by Modulating Mitochondrial Function in Aged Animal Skin. Antioxidants (Basel) 2023;12

5 Quiros PM, Goyal A, Jha P, Auwerx J: Analysis of mtDNA/nDNA Ratio in Mice. Curr Protoc Mouse Biol 2017;7:47-54.
